# Genome-wide Association Study of a Panel of Vietnamese Rice Landraces Reveals New QTLs for Tolerance to Water Deficit During the Vegetative Phase

**DOI:** 10.1186/s12284-018-0258-6

**Published:** 2019-01-28

**Authors:** Giang Thi Hoang, Lam Van Dinh, Thom Thi Nguyen, Nhung Kim Ta, Floran Gathignol, Chung Duc Mai, Stefan Jouannic, Khanh Dang Tran, Trung Huu Khuat, Vinh Nang Do, Michel Lebrun, Brigitte Courtois, Pascal Gantet

**Affiliations:** 1 National Key Laboratory for Plant Cell Biotechnology, Agricultural Genetics Institute, LMI RICE-2, Hanoi, 00000 Vietnam; 2 Genetic Engineering Division, Agricultural Genetics Institute, Hanoi, 00000 Vietnam; 3 University of Science and Technology of Hanoi, LMI RICE-2, Hanoi, 00000 Vietnam; 4 IRD, Université de Montpellier, LMI RICE-2, Hanoi, 00000 Vietnam; 5 IRD, Université de Montpellier, UMR DIADE, 34095 Montpellier, France; 6 IRD, Université de Montpellier, UMR LSTM, 34095 Montpellier, France; 7 Cirad, UMR-AGAP, F-34398 Montpellier, France; 8 CIRAD, INRA, Univ Montpellier, Montpellier SupAgro, Montpellier, France

**Keywords:** Association mapping, Drought tolerance, Genotyping by sequencing, Quantitative trait loci, Rice, Vietnamese landraces

## Abstract

**Background:**

Drought tolerance is a major challenge in breeding rice for unfavorable environments. In this study, we used a panel of 180 Vietnamese rice landraces genotyped with 21,623 single-nucleotide polymorphism markers to perform a genome-wide association study (GWAS) for different drought response and recovery traits during the vegetative stage. These landraces originate from different geographical locations and are adapted to different agrosystems characterized by contrasted water regimes. Vietnamese landraces are often underrepresented in international panels used for GWAS, but they can contain original genetic determinants related to drought resistance.

**Results:**

The panel of 180 rice varieties was phenotyped under greenhouse conditions for several drought-related traits in an experimental design with 3 replicates. Plants were grown in pots for 4 weeks and drought-stressed by stopping irrigation for an additional 4 weeks. Drought sensitivity scores and leaf relative water content were measured throughout the drought stress. The recovery capacity was measured 2 weeks after plant rewatering. Several QTLs associated with these drought tolerance traits were identified by GWAS using a mixed model with control of structure and kinship. The number of detected QTLs consisted of 14 for leaf relative water content, 9 for slope of relative water content, 12 for drought sensitivity score, 3 for recovery ability and 1 for relative crop growth rate. This set of 39 QTLs actually corresponded to a total of 17 different QTLs because 9 were simultaneously associated with two or more traits, which indicates that these common loci may have pleiotropic effects on drought-related traits. No QTL was found in association with the same traits in both the indica and japonica subpanels. The possible candidate genes underlying the quantitative trait loci are reviewed.

**Conclusions:**

Some of the identified QTLs contain promising candidate genes with a function related to drought tolerance by osmotic stress adjustment.

**Electronic supplementary material:**

The online version of this article (10.1186/s12284-018-0258-6) contains supplementary material, which is available to authorized users.

## Background

Rice is one of the most important crops for human nutrition, particularly in Asia where approximately 90% of the world’s rice production and consumption occur (FAO (Food and Agricultural Organization), 2001). Rice production is facing serious threats due to drought, extreme temperatures and other types of abiotic stresses linked to a lack of or excess water due to global climate change. In Vietnam, during the 2015–2016 period, a shortage of rainfall and heat occurred on a large scale in central coastal provinces, Central Highlands and south eastern regions, causing the worst drought over the last 90 years (Kantoush et al., 2017; Binh et al., 2017). In consequence, the drought caused significant damages to rice production by injuring plantlets at early vegetative development stage or by impairing seed production and yield. Drought is becoming a main limiting factor in rainfed rice areas of upland Vietnam. Therefore, there is an urgent need to create new varieties that are water-efficient and more tolerant to water deficits.

Drought tolerance is a complex quantitative trait controlled by many genes due to the intervention of many adaptive physiological and biochemical processes at both the cellular and plant levels with different effects at different stages of development, i.e., the seedling, vegetative or reproductive stages (Price and Courtois 1999; Tripathy et al. 2000; Xu et al. 2011; Yue et al. 2006; Nguyen and Bui 2008). Drought is particularly critical during the reproductive stage and often induces a yield reduction (O’Toole 1982; Price and Courtois 1999; Pantuwan et al. 2002; Yue et al. 2006; Kamoshita et al. 2008; Serraj et al. 2009, Todaka et al. 2015). A spikelet sterility of 73% was recorded when drought stress developed during the flowering stage (Cruz and O'Toole, 1984). A drought occurring during the vegetative stage had a moderate effect on subsequent plant development with a yield reduction of up to 30%, whereas during grain filling, a prolonged drought reduced grain yield by 75% (Boonjung and Fukai 1996). Nevertheless, at the tillering stage, drought stress reduces plant height, leaf length, and the number of tillers, and prolongs the vegetative stage (Rahman et al. 2002; Lanceras et al. 2004; Sarvestani et al. 2008; Venuprasad et al. 2009; Sudeshna et al. 2017). Sarvestani et al. (2008) also reported that a water deficit during the vegetative, flowering and grain filling stages reduced the grain yield by 21, 50 and 21%, respectively, in comparison to control, showing that globally, drought can affect yield regardless of the developmental stage.

Drought resistance mechanisms are often classified into four categories: drought escape through early maturity, drought avoidance through enhanced water uptake and reduced water loss, drought tolerance through osmotic adjustment, antioxidant capacity and desiccation tolerance, and drought recovery capacity (Price and Courtois 1999; Yue et al. 2006; Fang and Xiong 2015). Most agronomical research programs for drought tolerance have led to the identification of QTLs conferring better global water use efficiency and/or better osmoprotection (Fukai and Cooper 1995; Tuberosa and Salvi 2006).

In rice, several QTLs associated with drought resistance have been identified. This includes traits related to drought avoidance such as leaf water status maintenance (Price and Tomos 1997; Courtois et al. 2000; Yue et al. 2006), stomatal closure regulation (Price et al. 1997), and root morphology (Champoux et al. 1995; Yadav et al. 1997; Ali et al. 2000; Courtois et al. 2000; Zhang et al. 2001; Kamoshita et al. 2002; Price et al. 2002; Zheng et al. 2003; Yue et al. 2005; Courtois et al. 2013; Li et al. 2017). In rice, a deeper, thicker and more branched root system with a high root to shoot ratio can enhance the tolerance to water deficit in different environments (Gowda et al. 2011). This was recently well demonstrated with the discovery of the rice *DRO1* QTL that carries a gene impacting root gravitropism with a specific allele favoring a deeper root system. This allele is associated with an improved capacity to maintain yield under drought (Uga et al. 2013). Other QTLs associated with osmotic adjustment under drought have also been identified in rice. This concerns traits related to abscisic acid (ABA) content (Quarrie et al. 1997), cell membrane stability (Tripathy et al. 2000) or cell osmotic adjustment (Lilley et al. 1996; Zhang et al. 2001; Robin et al. 2003). Other QTLs associated with yield and yield-related traits (Muthukumar et al. 2015; Swamy et al. 2017), and drought recovery (Al-Shugeairy et al. 2015) have been reported. Most of these QTLs have been identified using bi-parental or multiparental populations, which have limited allelic diversity and poor resolution in QTL positioning (Korte and Farlow 2013; Swamy et al. 2017). More recently, genome-wide association studies (GWAS) provide opportunities to explore the enormous allelic diversity existing in natural populations and to position more precisely QTLs. GWAS offer the opportunity to scan collections of local rice landraces that have been selected for adaptation to adverse culture conditions and that often carry genes and alleles absent in modern varieties and that confer resistance to stresses, as for example *sub1* and *Pstol1* (Xu et al. 2006; Gamuyao et al. 2012). Some GWAS have been successfully carried out for drought tolerance-related traits and root traits in rice. Recently, a GWAS was carried out on a natural population consisting of 529 worldwide rice accessions for dissecting the genetic basis controlling 21 root traits under normal and drought stress conditions at the maturation stage (Li et al. 2017). Another GWAS, conducted on 75 Malaysia rice genotypes for yield and yield-related traits at the reproductive stage under drought stress, detected seven marker-trait associations for grain yield under drought stress (Swamy et al. 2017). The recent development of automated non destructive imaging systems have increased the capacity to phenotype larger panels. This allowed recently a robust detection by GWAS of many new QTLs involved in rice drought tolerance (Guo et al., 2018). Beside these genetic approaches the combination of transcriptomic and metablomic approaches allows to have a global understanding of the mechanisms that are involved in rice drought tolerance (Todaka et al., 2015, Todaka et al. 2017). This can help to identify the responsive genes in QTLs and to refine breeding strategies.

Vietnamese landraces are often underrepresented in panels used for GWAS, even in the fully sequenced 3 K genomes-panel (Li et al. 2014), while they can contain original genetic determinants related to drought resistance (Nguyen et al. 2006). In the present study, we used a panel of 180 Vietnamese rice landraces from different geographical locations and adapted to different agrosystems differing in water regimes (Phung et al. 2014). This panel was already successfully used to detect new QTLs related to root traits under well-watered conditions, such as the number of crown roots, deep root biomass and root thickness that could impact drought tolerance capacity (Phung et al. 2016). We evaluated the panel for drought tolerance at the vegetative stage with the objective of identifying QTLs valuable for increasing rice tolerance to water deficits. A large range of diversity was observed for leaf relative water content and drought sensitivity scores and several QTLs associated with these drought tolerance traits were identified by GWAS. Several genes with a functionlinked to osmotic stress signaling and adjustment andlikely related to drought tolerance have been identified inside the QTL confidence intervals and are discussed.

## Methods

### Plant Materials and Genotyping

A panel of 180 Vietnamese rice landraces and three genotyping controls (Nipponbare, IR64 and Azucena) were used as materials for the experiment (Additional file 1: Table S1). These Vietnamese traditional varieties differ by their geographical origin and by their watering regime (upland, rainfed lowland or irrigated). They were provided by the Plant Resource Center (Hanoi, Vietnam) and multiplied in the summer of 2014. This set of varieties included 113 indica, 64 japonica and 6 admixed accessions (controls included), according to the classification by Phung et al. (2014).

The panel was genotyped at 21,623 SNP markers that had a minor allele frequency (MAF) above 5%; among these markers, 13,814 and 8821 were polymorphic with the same MAF threshold in the indica and japonica subpanels, respectively (Phung et al. 2014). Additionally, 7 commercial Vietnamese rice varieties labelled Control1 to Control7 (RG1, KhangDan 18, QR9, BT7, Q5, RG10 and DS1) were used as phenotyping controls.

### Set up of the Phenotyping Experiment

The phenotyping experiment was conducted from June to August 2015 at the Van Giang experimental station (Lien Nghia commune, Van Giang district, Hung Yen province, Vietnam). The experiment was set in a nethouse fitted with a transparent plastic cover to protect it from rain. The design was an alpha-lattice design with 3 replicates. In each replicate, the accessions were distributed in 8 blocks of 24 plots, including two controls repeated two times (Additional file 1: Table S2).

Before the experiment, the rice seeds were incubated in an oven at 50 °C for 5 days to break the seed dormancy. Then, the fully germinated seeds were sown into 20x30x4 cm plastic trays filled with a thin layer of GT05 organic substrate, which contained 44% organic materials, 1.2% urea, 0.8% P_2_O_5_, and 0.7% K_2_O (*w*/w) (Research Center for Fertilizers and Plant Nutrients, Hanoi, Vietnam). After 1 week, 15 seedlings per accession were transplanted into 25*30*40 cm plastic pots filled with 10 kg of GT05 organic substrate. Five small holes for drainage were drilled at the bottom of each pot. Seven days after transplanting, thinning was conducted and 10 well-developed plants per pot were kept.

The plants were fully watered two times per day and plant protection methods were also applied to prevent pests and diseases. Four weeks after transplanting, the irrigation was stopped for 4 weeks and then resumed for another 2 more weeks before harvest.

### Sample Harvesting and Scoring

The first leaf sampling for relative water content (RWC) measurement was performed on the day before irrigation termination and recorded as T0. The four next harvests (T1 to T4) were carried out after each week of drought treatment. For this, a 7-cm-long leaf fragment was cut from the middle to the top of the second youngest fully expanded leaf and quickly put into a small zip plastic bag of known weight. The bags containing samples thereafter were weighed to determine the sample fresh weight (FW). Then, samples were put into 15-ml Falcon tubes containing 5 ml of distilled water overnight. The next day, the samples were taken out of the tube, the leaf surface was quickly dried with tissue paper, and the samples were immediately weighed again to determine the fully turgid weight (TW). The samples were then oven dried at 70 °C for 3 days and weighed to obtain the dry weight (DW). The RWC of each sampling was calculated as follows: RWC_Tn (%) = [(FW_Tn – DW_Tn)/(TW_Tn – DW_Tn)]*100 (*n* = 1 to 4, or ordinal number of samplings) (Turner 1981). The slope of the RWC after each week of drought stress (RWC_Sn) was computed as (RWC_Tn-1 – RWC_Tn)*100/RWC_Tn-1.

At the same time, from T1 to T4, a drought sensitivity score (Score1 to Score4) was visually recorded based on leaf rolling and drying using the standard evaluation system (SES) for rice (Additional file 1: Table S3) (IRRI 2002). To determine the recovery ability, the number of recovered plants was counted 2 weeks after rewatering. At T0 and T4, the shoot part of a plant was taken from each pot for determination of plant weight before stress (PlantW_bf) and after the 4-week stress (PlantW_aft). The relative crop growth rate (RCGR) was computed using the following formula: RCGR (%) = (PlantW_aft – PlantW_bf)*100/PlantW_bf. The traits measured with their abbreviations are listed in Additional file 1: Table S4.

### Statistical Analysis

Statistical analysis of phenotyping data was performed with R statistical software version 3.4.1. ANOVA was carried out to test the genotype effect. Broad-sense heritability (h^2^) was estimated to describe how each trait was affected by the environment.

In the whole panel as well as in the *indica* and *japonica* subpanels, Pearson’s correlations between monitored traits were calculated and displayed on corrplot using the R corrplot package. The significance of the results was tested by the function cor.test at the 0.95 confidence level. The adjusted means of the RWC_Tn, RWC_Sn, Scoren, RCGR and Recovery traits were used in GWAS.

### Genome-wide Association Study

The GWAS was conducted on the full panel and two subpanels (indica and japonica) using Tassel v.5.0 (Bradbury et al. 2007). We used a Mixed Linear Model (MLM) with control of kinship and population structure. The structure matrix was determined by a Principal Component Analysis (PCA) of the genotypic data. The number of axes to be kept was based on the analysis of the population structure, which had been previously conducted by Phung et al. (2014), i.e., six axes for the full panel, six for the indica subpanel and four for the japonica subpanel. To account for the relatedness among accessions of the panel, we established the kinship matrix using the Centered IBS method. Then, the GWAS results were presented as Q-Q plots to assess the quality of control of the number of false positives and as Manhattan plots based on the negative log10-transformed observed *p*-values for each SNP-trait association. We used a threshold of 5e-04 to declare a SNP significant.

### Linkage Disequilibrium (LD) Analysis

To allow us to determine the number of QTLs from all significant markers, we utilized the LDheatmap R package to produce a graphical display of pairwise linkage disequilibrium measures between SNPs in the genomic regions where significant SNPs (*P* < 1e-04) were located. The QTL intervals were limited to regions where the r^2^ values (squared allele frequency correlation) between markers were above 0.4. In case the identified LD block around significant marker(s) was less than 50 kb, we expanded the QTLs up to 50 kb upstream and downstream of the detected regions.

### Identification of Candidate Genes

In an attempt to extract the genes underlying the QTLs of interest, we scanned the genome regions of these QTLs in the MSU rice database. Next, from the gene lists, the candidate genes were identified based on the predicted function (biological processes) or expression pattern of genes in relation with the trait of interest.

## Results

### Phenotypic Variation and Heritability Analysis Showed that the Panel Globally Presented an Homogeneous Response to Drought and Exhibited Variability at the Genotype Level

**Fig. 1:**
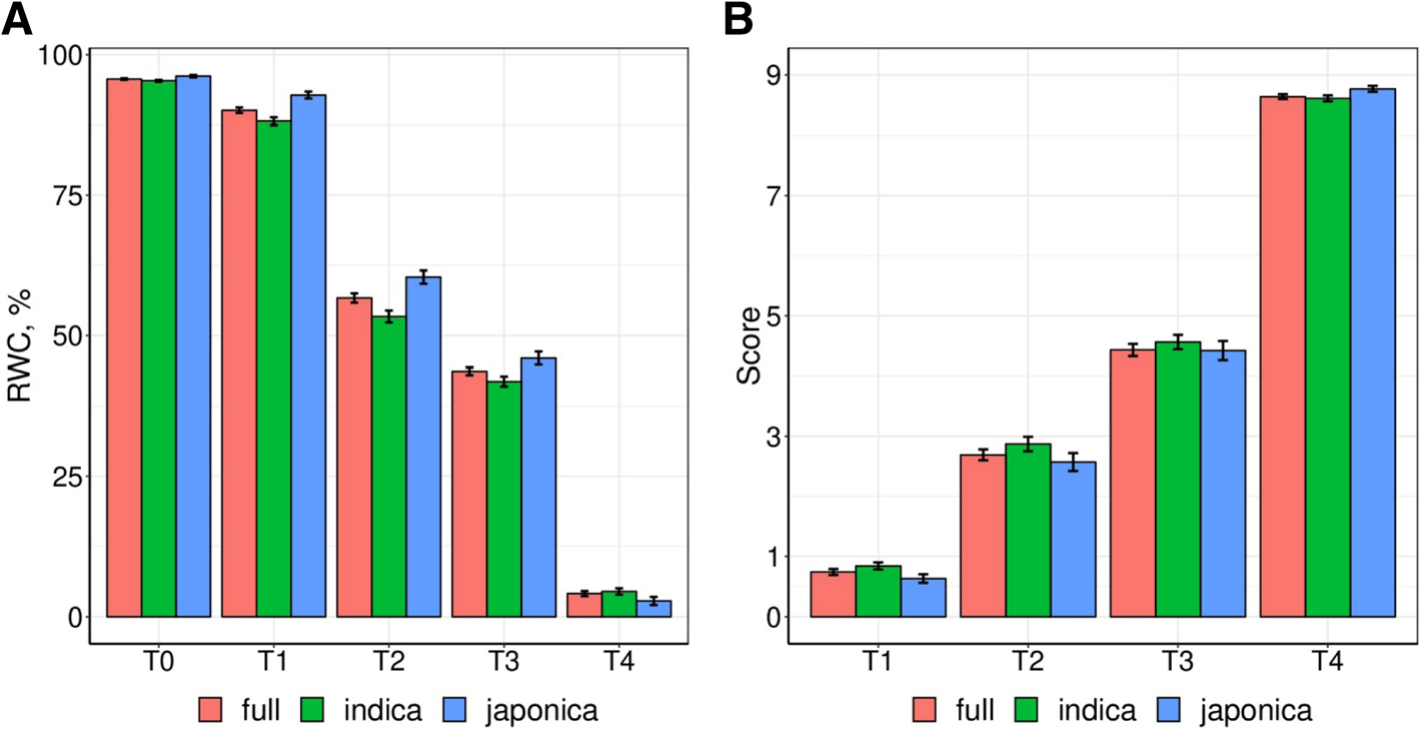
Variation of relative water content in leaf (**a**) and SES score (**b**) from 0 to 4 weeks of drought stress according to the three panels. RWC, relative water content; Score, drought sensitivity score; T0, the day before drought treatment; T1 to T4, one to four weeks of drought stress

To assess the severity level of the stress in our trial, we analyzed the variation of the average RWC and Score of the panel over time (from T1 after 1 week of stress to T4 after 4 weeks of stress). The average RWC decreased from approximately 96% before stress to 4% after 4 weeks of drought stress, while the average Score increased from 0.7 at T1 to 8.6 at T4 (Additional file 1: Table S5). These values indicate that a severe drought stress was successfully created in this experiment. Figure 1 shows that for both traits, the variation was not linear overtime. The slope was steeper between T1 and T2 and T3 and T4 for RWC, and between T3 and T4 for Score. Figure 1 also shows that the effect of the drought stress on RWC and on Score was similar for the three panels.

ANOVA showed that the variety effect was statistically significant for all of the traits, with the exception of the traits monitored at T0 or at T1, at a time where the stress was no yet fully established, and for RCGR (Table 1). For the traits with a significant variety effect, broad-sense heritability (h^2^) ranged from 0.15 to 0.64. The mean, standard deviation, maximum value, minimum value and coefficient of variation (CV) of all selected traits for the full panel are shown in Additional file 1: Table S5. A large range of variation was observed, with CVs of the panel varying from 19.7 to 154.7% for most of the traits, apart for the relative water content before or after 1 week of drought treatment (RWC_T0 and RWC_T1), the slope of relative water content after 4 weeks of drought stress (RWC_S4) and the drought sensitivity score after 4 weeks of drought stress (Score4), which had a lower CV.

**Table 1:** Variance analysis and trait broad sense heritability

Trait	Rep	Accession	h^2^
RWC_T0	< 0.001	0.2214	0.15
RWC_T1	< 0.001	0.1721	0.18
RWC_T2	0.6497	0.0046	0.43
RWC_T3	0.0954	0.0322	0.33
RWC_T4	0.0011	0.0349	0.32
RWC_S1	< 0.001	0.4175	0.05
RWC_S2	0.1124	0.0343	0.33
RWC_S3	0.1308	0.6601	0.00
RWC_S4	0.0010	0.0242	0.34
Score1	0.1425	0.4848	0.01
Score2	0.2900	0.0907	0.25
Score3	0.7322	0.0312	0.33
Score4	0.0061	0.0349	0.32
Recovery	0.2361	< 0.001	0.64
RCGR	0.1593	0.6184	0.00

*Rep* replication, *RWC_T0* relative water content before drought treatment, *RWC_T1 to RWC_T4* relative water content after 1 to 4 weeks of drought stress, *RWC_S1 to RWC_S4* slope of relative water content after 1 to 4 weeks of drought stress, *Score1 to Score4* drought sensitivity score after 1 to 4 weeks of drought stress, *Recovery* recovery ability, *RCGR* relative crop growth rate

**Fig. 2:**
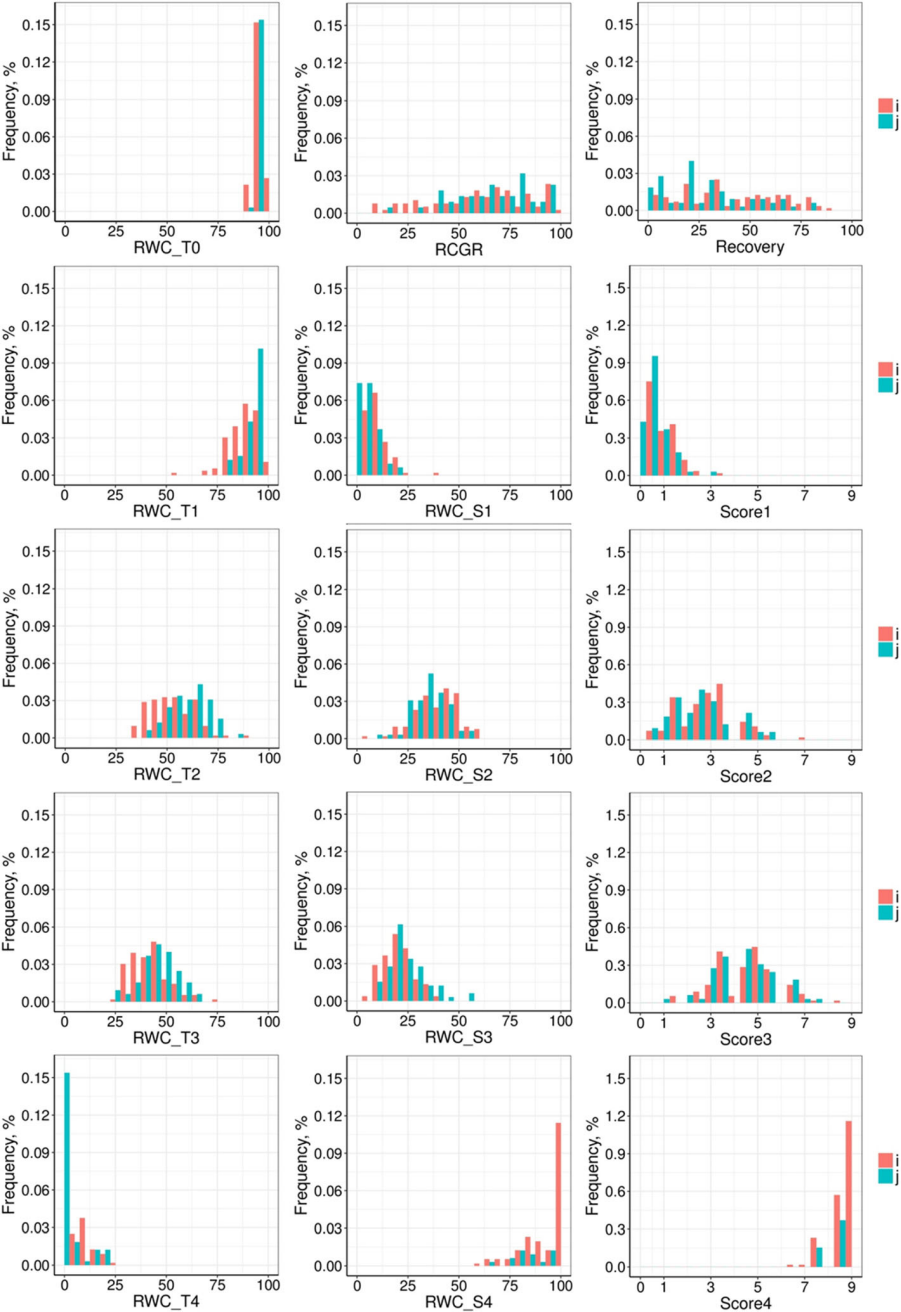
Frequency distribution of calculated traits. Indica subpanel (I) in red; japonica (J) subpanel in blue; RWC_T0, relative water content before drought treatment; RWC_T1 to RWC_T4, relative water content after 1 to 4 weeks of drought stress; RWC_S1 to RWC_S4, slope of relative water content after 1 to 4 weeks of drought stress; Score1 to Score4, drought sensitivity score after 1 to 4 weeks of drought stress; Recovery, recovery ability; RCGR, relative crop growth rate

The same elements for the indica and japonica subpanels are presented in Table 2. During the drought treatment, the japonica accessions had on average a higher leaf RWC and lower Score than the indica accessions, with the exception of T4, whose values of RWC and Score were slightly lower and greater, respectively, than for the indica subpanel. Consequently, the indica accessions had a steeper slope of RWC during the first 2 weeks of drought stress (RWC_S1 and RWC_S2), then a flatter slope in the third and the fourth weeks. On average, the indica accessions had a lower RCGR during the stress but a better recovery ability than the japonica accessions. However, the trait distributions of the indica and japonica subpanels largely overlapped (Fig. 2). Overall, these results reflect inherent differences in drought tolerance between the subpanels, with a greater drought-tolerance of the japonica subpanel during the first 3 weeks of stress. However, after 3 weeks, the two subpanels were equally affected (Additional file 2: Figure S1).

**Table 2:** Descriptive statistics of the indica (ind) and japonica (jap) subpanels for selected traits

Traits	*n*	*n*	mean	sd	min	max	CV	mean	sd	min	max	CV
	ind	jap	ind	ind	ind	ind	ind	jap	jap	jap	jap	jap
RWC_T0 (%)	112	65	95.3	2.0	90.6	99.9	2.1	96.2	1.7	92.3	99.8	1.8
RWC_T1 (%)	112	65	88.2	7.2	54.9	99.2	8.2	92.8	5.0	78.7	99.1	5.3
RWC_T2 (%)	104	65	53.4	10.9	34.3	88.6	20.4	60.4	9.6	39.8	86.9	15.8
RWC_T3 (%)	112	65	41.9	9.5	25.8	73.8	22.6	46.0	9.3	23.6	66.6	20.2
RWC_T4 (%)	112	65	4.4	6.1	0.0	23.5	136.3	2.7	5.8	0.0	21.7	212.8
RWC_S1 (%)	112	65	8.8	6.6	0.0	39.7	74.9	4.9	4.6	0.0	18.9	95.1
RWC_S2 (%)	104	65	40.1	10.8	7.0	62.3	27.0	35.1	8.8	10.6	55.4	25.1
RWC_S3 (%)	104	65	20.5	7.6	5.5	39.4	37.2	24.4	9.4	9.6	54.9	38.5
RWC_S4 (%)	112	65	92.9	10.1	59.8	100.0	10.8	96.3	8.1	62.7	100.0	8.4
Score1	112	65	0.8	0.7	0.0	3.7	78.6	0.6	0.6	0.0	3.0	92.1
Score2	112	65	2.9	1.2	0.3	7.0	42.9	2.6	1.2	0.7	5.7	48.3
Score3	112	65	4.6	1.3	1.3	8.3	28.3	4.4	1.3	1.0	7.7	29.4
Score4	112	65	8.6	0.5	6.3	9.0	6.2	8.8	0.4	7.7	9.0	4.7
Recovery (%)	112	65	39.9	24.7	0.0	88.9	61.9	28.5	21.1	0.0	77.8	74.2
RCGR (%)	109	65	83.6	46.8	10.3	296.3	55.9	89.7	41.9	13.6	219.5	46.7

*RWC_T0* relative water content before drought treatment, *RWC_T1 to RWC_T4* relative water content after 1 to 4 weeks of drought stress, *RWC_S1 to RWC_S4* slope of relative water content after 1 to 4 weeks of drought stress, *Score1 to Score4* drought sensitivity score after 1 to 4 weeks of drought stress, *Recovery* recovery ability, *RCGR* relative crop growth rate

**Fig. 3:**
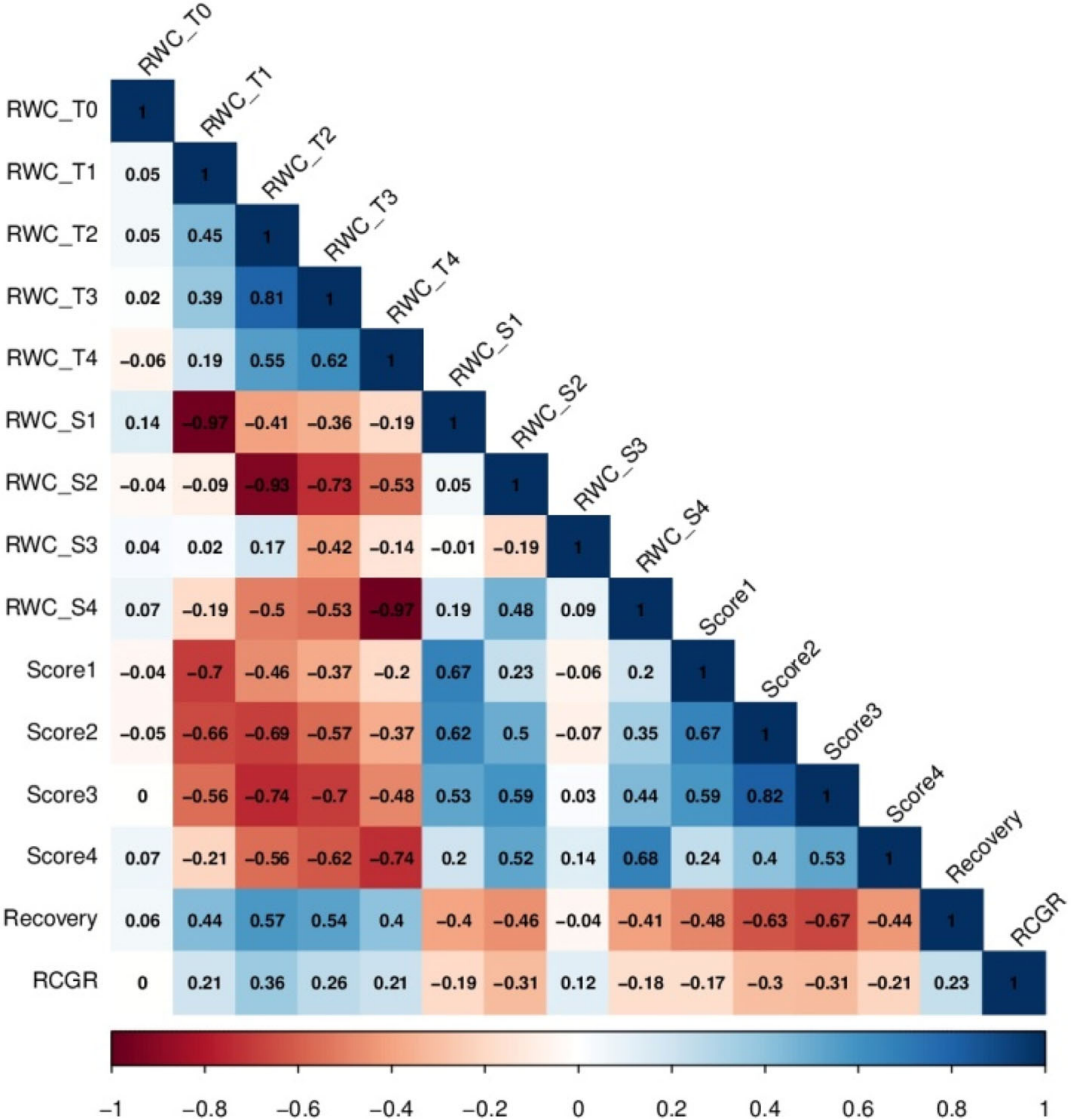
Correlations between traits in the full panel. RWC_T0, relative water content before drought treatment; RWC_T1 to RWC_T4, relative water content after 1 to 4 weeks of drought stress; RWC_S1 to RWC_S4, slope of relative water content after 1 to 4 weeks of drought stress; Score1 to Score4, drought sensitivity score after 1 to 4 weeks of drought stress; Recovery, recovery ability; RCGR, relative crop growth rate

By analyzing the correlation among the traits, we observed that most of the traits were moderately correlated with each other (Additional file 1: Table S6, Fig. 3 and Additional file 3: Figure S2), except for RWC_T0, whose correlation coefficients r were < ±0.15. The absence of a significant correlation with RWC_T0 can easily be explained by the fact that the stress was not initiated at T0. Within the full panel and the two subpanels, similar trends were observed, even though tiny variations occurred from trait to trait (Additional file 1: Table S6). As expected in these longitudinal data, the correlations between measurements made on the same dates (RWC_T, RWC_S or Score) were the highest, followed by correlations between measurements made at successive dates, with an exception for RWC_S3 for which all correlations were weak. For Recovery, the correlation was at its highest with Score3 (− 0.67 in the full panel). RCGR was modestly correlated with the other traits (less than ±0.36 in the full panel).

### A Genome-wide Association Study Reveals QTLs Associated with Drought Tolerance Traits

**Fig. 4:**
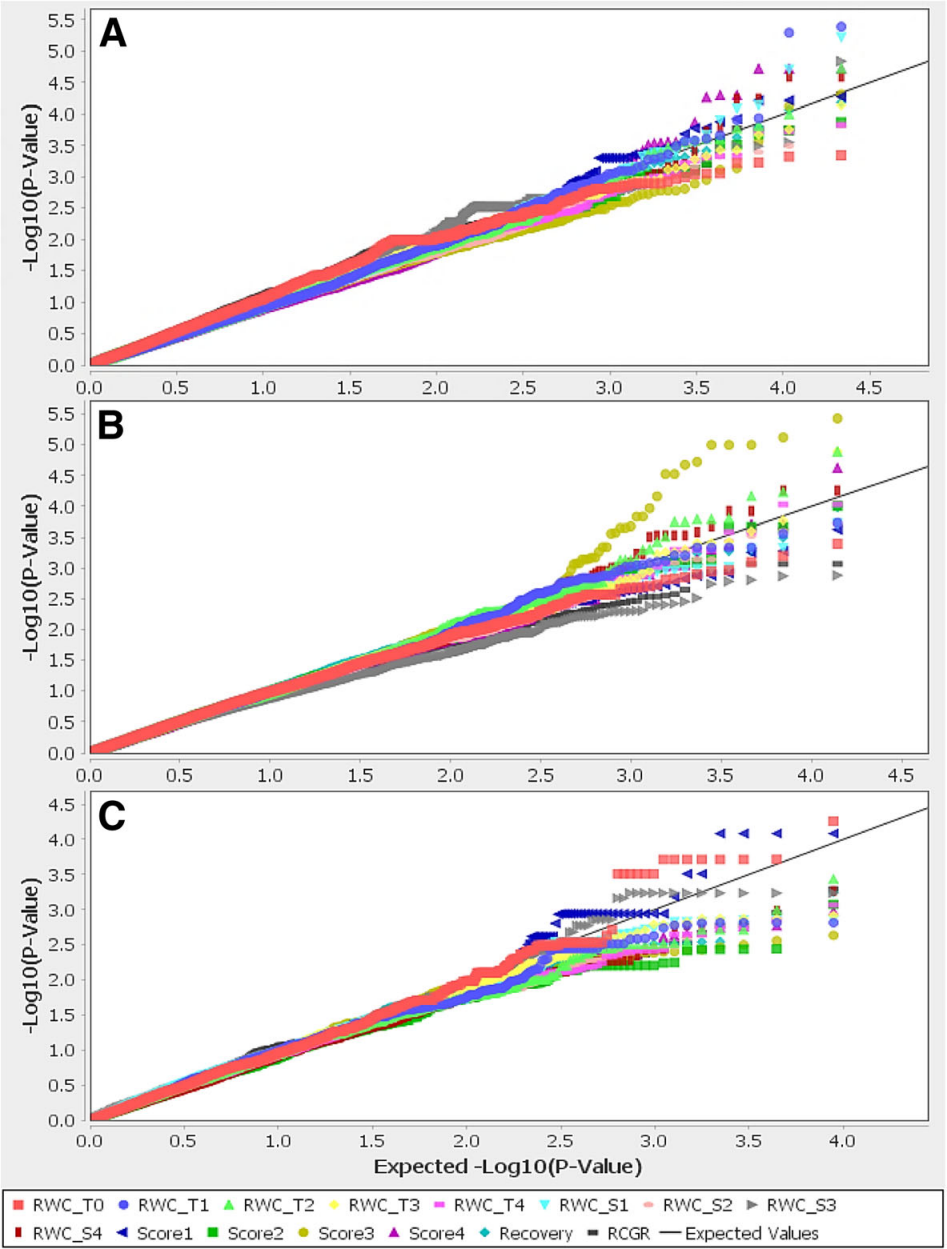
Quantile-quantile plots for the full panel (**a**) and the indica (**b**) and japonica (**c**) subpanels. RWC_T0, relative water content before drought treatment; RWC_T1 to RWC_T4, relative water content after 1 to 4 weeks of drought stress; RWC_S1 to RWC_S4, slope of relative water content after 1 to 4 weeks of drought stress; Score1 to Score4, drought sensitivity score after 1 to 4 weeks of drought stress; Recovery, recovery ability; RCGR, relative crop growth rate

**Fig. 5:**
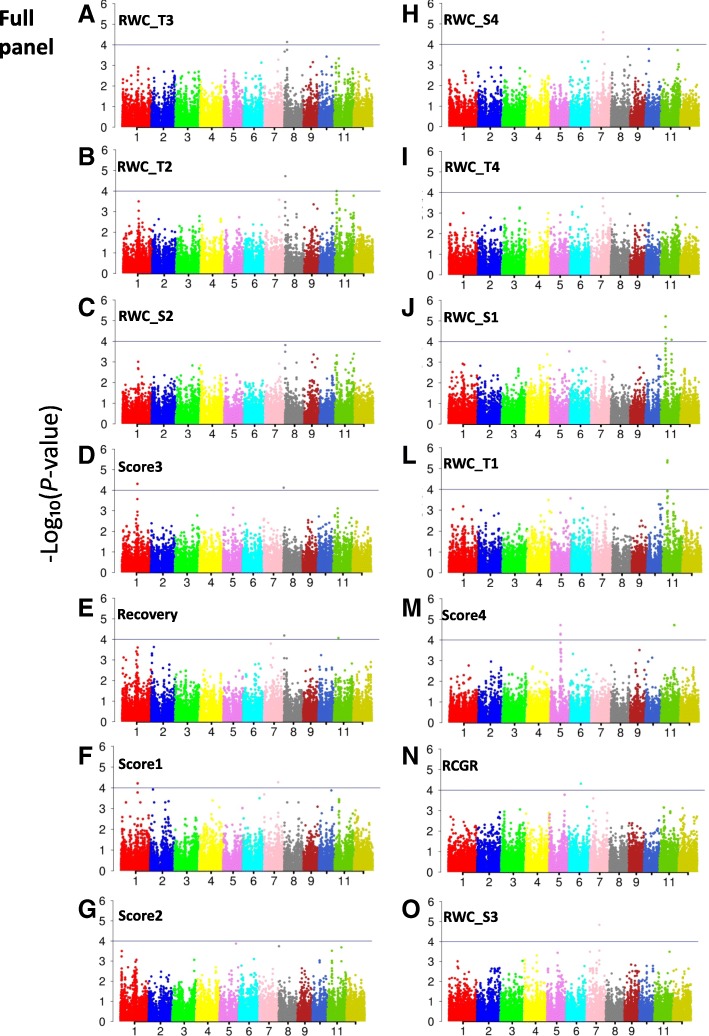
Manhattan plots for genome-wide association of drought-related traits with SNP markers for the full panel. RWC_T1 to RWC_T4, relative water content after 1 to 4 weeks of drought stress; RWC_S1 to RWC_S4, slope of relative water content after 1 to 4 weeks of drought stress; Score1 to Score4, drought sensitivity score after 1 to 4 weeks of drought stress; Recovery, recovery ability; RCGR, relative crop growth rate

To identify genomic regions associated with the measured traits, we carried GWAS for the full panel and, then, for the indica and japonica subpanels. To overcome false positive associations, we used a mixed linear model involving kinship and structure as applied in Phung et al. (2016) who used the same rice panel. On the QQ plots of all traits (Fig. 4), the observed *P*-values followed a uniform distribution and obviously deviated from the expected *P*-value distribution only in the upper tail of the diagonal line. Therefore, the model appears suitable for association mapping with this dataset as well. The results of the association analyses are reported using Manhattan plots in Fig. 5 for the full panel and in Additional file 4: Figure S3 for the indica and the japonica subpanels.

When fixing a threshold of 5e-04 to declare an association, a total of 77, 59 and 6 SNPs-trait associations were detected that corresponded to 49, 32 and 6 significant SNPs detected in the full panel, and the indica and japonica subpanels, respectively (Table 3). Thus, many SNPs detected as significant in the full panel were also significant in the two subpanels. Notably, all 87 significant SNPs were tagged to only 17 genomic regions, which were considered as QTLs, distributed on chromosomes 1, 2, 5, 6, 7, 8, 10 and 11. Of these 17 QTLs, 10 were detected both in the full panel and in the indica subpanel, and one was found both in the full panel and in the japonica subpanel. Among the 6 remaining QTLs, four (q5, q6, q7 and q8) were identified only in the full panel, one (q11 for Score4) only in the indica subpanel, and one (q3 for RWC_T0) in the japonica subpanel. The much higher number of QTLs detected in the indica subpanel than in the japonica subpanel is most likely linked to the larger size of the indica subpanel. Our results are similar with those of Phung et al. (2016) using the same Vietnamese rice panel who also found more QTLs in the indica subpanel, with no significant association shared by the indica and the japonica subpanels.

**Table 3:** QTLs common across traits for the three panels. A – full panel; B – indica subpanel; C – japonica subpanel (*P*-values of the QTLs detected as significant at P < 5e-04)

QTLs	Chr	Position	Nb ass.	RWC_T0	RWC_T1	RWC_T2	RWC_T3	RWC_T4	RWC_S1	RWC_S2	RWC_S3	RWC_S4	Score1	Score2	Score3	Score4	Recovery	RCGR
A. Full panel																		
q1	1	23,006,901	1			3.2E-04												
		23,083,234	2												4.9E-05		2.5E-04	
q2	1	24,510,022	1												1.7E-04			
		24,533,617	1												6.1E-05			
		24,563,013	1												6.1E-05			
q4	5	15,011,955	1													1.9E-05		
		15,023,372	1													5.4E-05		
		15,034,791	1													3.8E-04		
		15,064,145	1													5.1E-05		
		15,064,148	1													5.1E-05		
		15,089,160	1													2.8E-04		
		15,092,328	1													2.8E-04		
		15,152,887	1													2.9E-04		
		15,555,144	1													2.9E-04		
		15,756,028	1													2.8E-04		
		15,801,387	1													4.4E-04		
q5	6	17,849,604	1															4.9E-05
q6	7	17,904,510	1									5.7E-05						
		17,904,513	2					4.7E-04				5.7E-05						
		17,947,305	2					1.9E-04				2.5E-05						
		17,947,308	2					1.9E-04				2.5E-05						
q7	7	19,695,647	1								1.5E-05							
q8	7	20,730,455	1										5.4E-05					
		20,791,018	1			2.6E-04												
q9	8	298,529	2			3.4E-04				3.2E-04								
		415,144	6			1.9E-05	2.1E-04			1.5E-04				1.8E-04	7.5E-05			6.5E-05
q10	8	4,048,851	1				1.8E-04											
		4,053,013	1				7.2E-05											
q12	11	2438,402	1			4.1E-04												
		2,440,016	2			1.5E-04				4.9E-04								
		2,441,445	1			4.1E-04												
		2,444,894	2			1.0E-04				4.8E-04								
		2,444,897	1			2.8E-04												
		2,445,085	1			2.5E-04												
		2,446,180	1			2.5E-04												
q13	11	6,647,262	3		5.1E-06				2.0E-05				4.9E-04					
q13*	11	6,662,124	3		2.5E-04								3.7E-04	3.1E-04				
		6,691,388	3		2.6E-04				2.8E-04				4.2E-04					
		6,691,391	3		4.4E-04				4.6E-04				3.5E-04					
		6,706,811	4		1.2E-04		4.6E-04		2.2E-04								8.5E-05	
		6,887,831	3		1.2E-04				1.3E-04				3.9E-04					
q14	11	6,934,618	2		4.1E-06				6.0E-06									
q15	11	7,603,100	2		2.2E-04				7.2E-05									
q16	11	15,692,357	1						4.0E-04									
		15,692,360	1						4.0E-04									
		15,712,256	1						4.0E-04									
		15,796,210	2		4.9E-04				8.2E-05									
q17	11	19,980,023	1													1.9E-05		
		20,322,700	1													1.9E-05		
*B. Indica* subpanel																		
q1	1	22,974,974	1												1.4E-05			
		23,006,901	2												2.4E-05		4.0E-04	
		23,083,234	2												2.4E-05		4.0E-04	
		23,151,112	2												2.4E-05		4.0E-04	
		23,168,543	1												6.6E-05			
		23,351,413	2			4.9E-04				3.6E-04								
q4	5	15,011,955	1													1.6E-04		
		15,023,372	1													3.1E-04		
		15,064,145	1													3.8E-04		
		15,064,148	1													3.8E-04		
q6	7	17,904,510	2					2.3E-04				1.3E-04						
		17,904,513	2					2.3E-04				1.3E-04						
		17,912,789	1									2.8E-04						
		17,947,305	2					8.3E-05				6.4E-05						
		17,947,308	2					8.3E-05				6.4E-05						
q9	8	298,529	1			3.1E-04												
		415,144	6			1.3E-05	5.6E-06			1.9E-04					4.6E-06	3.5E-04	5.6E-05	
q11	10	10,281,766	1													1.8E-05		
q12	11	2438,402	1			1.8E-04												
		2,440,016	3			6.8E-05	1.4E-04			2.5E-04								
		2,441,445	1			1.8E-04												
		2,444,894	4			5.9E-05	1.1E-04			3.0E-04					9.8E-05			
		2,444,897	2			1.6E-04									2.1E-04			
		2,445,085	3			1.6E-04	2.2E-04								9.0E-05			
		2,446,177	2							3.1E-04					2.5E-04			
		2,446,180	3			1.6E-04	2.2E-04								9.0E-05			
q13	11	6,647,262	3		4.6E-05		4.2E-04		1.3E-04									
q14	11	6,934,618	2		6.0E-05				6.3E-05									
q15	11	7,603,100	1						3.5E-04									
q16	11	15,796,210	1						3.8E-04									
q17	11	19,980,023	1													8.2E-05		
		20,322,700	1													8.2E-05		
*C. Japonica* subpanel																		
q2	1	24,502,190	1										8.3E-05					
		24,510,022	1										8.3E-05					
		24,533,617	1										8.3E-05					
		24,563,013	1										8.3E-05					
q3	2	35,019,983	1	1.9E-04														
		35,082,166	1	5.5E-05														

*RWC_T0* relative water content before drought treatment, *RWC_T1 to RWC_T4* relative water content after 1 to 4 weeks of drought stress, *RWC_S1 to RWC_S4* slope of relative water content after 1 to 4 weeks of drought stress, *Score1 to Score4* drought sensitivity score after 1 to 4 weeks of drought stress, *Recovery* recovery ability, *RCGR* relative crop growth rate

For each QTL, the number of SNPs ranged from 1 to 11, with the highest number of SNPs found in q4 for Score4 (Table 4). Nine of the 17 QTLs were commonly detected for two or more traits. The most striking is QTL q9 common to 7 traits, including RWC_T2, RWC_T3, RWC_S2, Score2, Score3, Score4 and Recovery. Except for RWC_T0, for which the initial ANOVA did not detect variation between varieties (Table 1), a total of 13 QTLs for Relative Water Content were identified, four for RWC_T1, four for RWC_T2, four for RWC_T3, and one for RWC_T4 (Additional file 1: Table S7). A common significant region was observed for RWC_T1 and RWC_T3 (q13), and two other ones for RWC_T2 and RWC_T3 (q9 and q12). Remarkably, we found four significant SNPs on chromosome 11 (q12) associated with RWC_T2 that were also recorded to be associated with RWC_T3 across the indica subpanel. For the slope of relative water content, RWC_S1, RWC_S2 and RWC_S4 nearly detected the same QTLs as those detected for RWC_T1, RWC_T2 and RWC_T4, respectively. Similarly, there was a substantial overlap between the QTLs detected for score traits. For example, q13 was shared between Score1 and Score2, and q9 between Score2, Score3 and Score4. However, only one single QTL per trait was detected for RCGR and RWC_S3 (q5 and q7, respectively in Additional file 1: Table S7), which can be explained by the low heritability of these traits and their weak phenotypic correlation with the other traits (Fig. 3). Interestingly, a total of 22 significant SNPs mapped to chromosome 11 for RWC, RWC_S and Score, which were clustered into 6 LD blocks (i.e., q12 to q17) (Table 4). Among them, q14 and q15 were defined by a single significant marker within a low LD block. The QTL with the strongest associations (q14, *P* < 10^− 5^) was detected on chromosome 11 for both RWC_T1 and RWC_S1 in the full panel, and explained 12.7% and 12.3% of the phenotypic variation, respectively (Additional file 1: Table S7). Likewise, in the indica subpanel, a common significant SNP (q9, *P* < 10^− 5^) for Score3 and RWC_T3 was identified on chromosome 8 to have a large effect with a contribution of 16.7–17.1% to the phenotypic variation. Additionally, there was a highly significant SNP (tagged to q13, *P* = 5.1e-06) for the full panel on chromosome 11, which accounted for 12.4% of the phenotypic variation for RWC_T1.

**Table 4:** List of candidate genes located within the identified QTLs

QTL name	Chr	QTL position	Traits	No of signif.SNPs	Gene ID	Gene annotation	References
q1	1	22974974–23324354	RWC_T2, RWC_S2, Score3, Recovery	6	*LOC_Os01g40860*	*OsALDH2–1*, aldehyde dehydrogenase	
					*LOC_Os01g40870*	*OsALDH2–2*, aldehyde dehydrogenase	Ma et al., 2016
q2	1	24234676-24848754	Score1	4	*LOC_Os01g42850*	OsATG7, postmeiotic anther development	Kurusu et al., 2015
q3	2	35019983-35135670	RWC_T0	2	*LOC_Os02g57250*	OsIAA10, auxin-responsive Aux 2FIAA gene	
q4	5	14993554-15948192	Score4	11	*LOC_Os05g25770*	*OsWRKY45*, transcription factor	Tao et al., 2011
					*LOC_Os05g25850*	*OsMSOD1*, superoxide dismutase	de Deus et al., 2015; Song et al., 2014
q5	6	17773531-17849604	RCGR	1			
q6	7	17904510-18115227	RWC_T4, RWC_S4	5	*LOC_Os07g30590*	*OsLTPG20*, LTPL55 - protease inhibitor seed storage LTP family protein precursor	Edstam et al., 2013
q7	7	19592999-19695647	RWC_S3	1			
q8	7	20593830-20792416	RWC_T2, Score1	2			
q9	8	298529-415144	RWC_T2, RWC_T3, RWC_S2, Score2, Score3, Score4, Recovery	2			
q10	8	3775479-4300868	RWC_T3	2	*LOC_Os08g07440*	AP2/EREBP68, transcription factor	
					*LOC_Os08g07060*	OsCRR6, photosynthetic capacity	Yamori et al., 2011
q11	10	10281766	Score4	1			
q12	11	2438402-2567319	RWC_T2, RWC_T3, RWC_S2, Score3	8	*LOC_Os11g05470*	*OsRCN1*, PEBP family protein, negative response to cold, salinity, and heat stress	Tripathi et al., 2012
					*LOC_Os11g05480*	*OsbZIP79*, transcription factor HBP-1b, dehydration and salt stress response	Nijhawan et al., 2008
					*LOC_Os11g05640*	*OsbZIP80*, transcription factor, dehydration stress response	Nijhawan et al., 2008
q13	11	6642371-6895086	RWC_T1, RWC_T3, RWC_S1, Score1, Score2, Recovery	6		NBS-LRR proteins, disease resistance	Magbanuaet al., 2014; Onaga, 2014; Hou et al., 2015
q14	11	6934618-7049977	RWC_T1, RWC_S1	1	*LOC_Os11g12530*	Protein kinase, drought stress response	Chandran et al., 2016
q15	11	7603100	RWC_T1, RWC_S1	1	*LOC_Os11g13840*	OsERF19, AP2 domain containing protein	
q16	11	15672814-16164499	RWC_T1, RWC_S1	4	*LOC_Os11g27329*	OsSCP61, peptidase S10	
q17	11	19956781-20322700	ScorT4	2			

*RWC_T0* relative water content before drought treatment, *RWC_T1 to RWC_T4* relative water content after 1 to 4 weeks of drought stress, *RWC_S1 to RWC_S4* slope of relative water content after 1 to 4 weeks of drought stress, *Score1 to Score4* drought sensitivity score after 1 to 4 weeks of drought stress; *Recovery* recovery ability, *RCGR* relative crop growth rate

The identified associations were compared to the 442 QTLs for drought-related traits (root traits excluded) detected in mapping populations under stress conditions retrieved from the QTL module TropgeneDB (http://tropgenedb.cirad.fr/tropgene/JSP/interface.jsp?module=RICE). The 22 QTLs whose confidence interval overlapped with an association detected in this study are listed in Additional file 1: Table S8. In addition to the co-locations with QTLs deriving from mapping populations, we also observed overlaps between the associations from our study and the associations for drought-related traits detected using genome-wide association mapping (Courtois et al. 2013; Muthukumar et al. 2015; Al-Shugeairy et al. 2015; Swamy et al. 2017, Guo et al. 2018). It found only 58 overlaps in a total of 1889 GWAS sites collected, in which there are 31 associations from the present study underlying the QTLs q1, q4, q5, q7, q9, q11, q13, and q14 (Additional file 1: Table S9). When comparing the map positions of the QTLs detected in the present study with those of the QTLs identified by Phung et al. (2016) for root traits using the same rice germplasm collection and genotyping data, no overlap was found (Additional file 1: Table S9, Additional file 5: Figure S4).

### Scanning Candidate Genes Inside QTL Regions Reveals Stress Response- and Osmotic Adjustment-related Genes

**Fig. 6:**
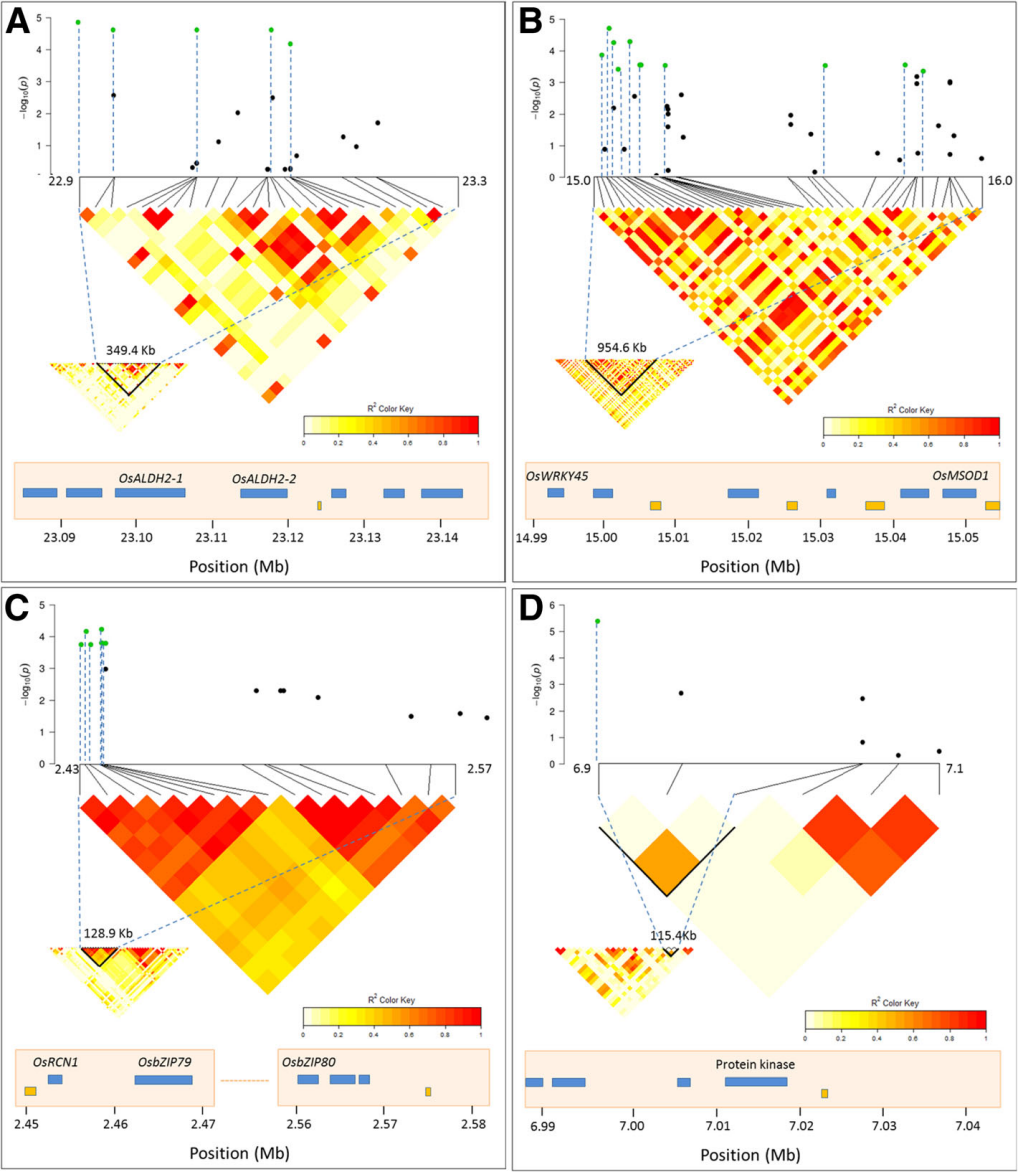
Manhattan plots and Linkage Disequilibrium (LD) heatmaps for some strong QTLs. **a** Score3 across q1 in the indica subpanel; **b** Score4 across q4 in the full panel; **c** RWC_T2 across q12 in the indica subpanel; **d** RWC_T1 across q14 in the full panel. The well-known genes of interest in these regions are also illustrated

Among the 17 identified QTLs, we focused on the seven QTLs (q1, q4, q7, q9, q13, q12, and q14) having the highest significance or which were detected several times for different traits. Then, the genes in the QTL regions were investigated by using the *Oryza sativa* reference genome database and bibliography survey to search for candidates related to drought tolerance. QTL q1 contained 6 strong SNPs detected for four traits (RWC_T2, RWC_T3, Score3 and Recovery) and was located from 22,975.2 to 23,324.6 Kb (349.4 Kb) on chromosome 1. Across q1, we found two interesting genes annotated as drought-tolerance-related, which were *OsALDH2–1* (*LOC_Os01g40860*) and *OsALDH2–2* (*LOC_Os01g40870*) (Fig. 6a). QTL q4 with the largest cluster of significant SNPs was mapped on chromosome 5 for Score4 in the full panel (Table 4). Within this locus (q4, 954.6 Kb), we found two candidate genes already known to control drought tolerance, namely, *OsWRKY45* (*LOC_Os05g25770*) (Tao et al. 2011) and *OsMSOD1* (*LOC_Os05g25850*) (Song et al. 2014; de Deus et al., 2015) (Fig. 6b). On chromosome 7, we identified a strong peak of 5 significant SNPs (q6) for RWC_S4 (Fig. 5h, Additional file 4: Figure S3 H indica), which co-localized with the peak for RWC_T4 (Fig. 5i, Additional file 4: Figure S3 I indica), and covered 210.7 Kb (17,904.5–18,115.2 Kb). However, in this region, no gene has been predicted to be associated with drought stress response. On chromosome 8, QTL q9 was associated with 7 different traits (Table 4) but, inside this region (298.5–415.1 Kb) no candidate gene related to drought tolerance was identified. QTL q13 on chromosome 11, which was associated with 6 traits, including RWC_T1, RWC_T3, RWC_S1, Score1, Score2, and Recovery (Table 4), ranged from 6642.4 to 6895.1 Kb, but similarly to q9, this region did not include any good candidate gene either. QTL q12 involving 8 significant SNPs on chromosome 11 and associated with four traits (i.e. RWC_T2, RWC_T3, RWC_S2, and Score3), ranged from 2438.4 to 2567.3 Kb (interval of 128.9 Kb; Fig. 6c). In this region, three genes were reported to have a role in or to be differentially regulated during drought-stress response: *OsRCN1* (*Os11g05470*) (Tripathi et al. 2012), *OsbZIP79* (*Os11g05480*) and *OsbZIP80* (*Os11g05640*) (Nijhawan et al. 2008). Lastly, QTL q14 exhibited a strong association with RWC_T1 for the full panel (Table 4) and was located within a 115.4 Kb interval on chromosome 11 (Fig. 6d) that includes a gene previously identified by transcriptome meta-analysis to be associated with drought stress response (*Os11g12530*) (Chandran et al. 2016). All of this information is presented in Table 4.

## Discussion

Drought tolerance is controlled by a large number of traits and genes. In rice, so far, few association studies have been conducted for drought-tolerance related traits other than root system architecture (Vannirajan et al. 2012; Muthukumar et al. 2015; Al-Shugeairyet al. 2015; Swamy et al. 2017) and many of them have focused on the yield impact of drought during the reproductive stage. The present study is a genome-wide association study focusing on drought tolerance at the vegetative stage. In this study, a panel of 180 Vietnamese rice landrace accessions was phenotyped during and after a 4-week drought stress. The kinetics of leaf relative water content and drought sensitivity score was estimated throughout the drought stress treatment. The panel coefficients of variation clearly confirmed a large variation of traits in response to drought stress. A high broad-sense heritability was observed for almost all primary traits, except for the plant weight before and after drought stress. For the secondary traits that were calculated from the primary traits and that were used in the GWAS, heritability was diminished, which is commonly observed due to the propagation of uncertainty in functions involving several variables (Table 1). The phenotypic correlations between traits showed that RWC_T and Score were highly correlated with negative correlation coefficients, and that these correlations were at their highest when the measurements were recorded on the same date. These results confirm that drought sensitivity score, i.e. leaf rolling and leaf drying, was associated with declining leaf water status. Similar results were also reported in the context of the doubled-haploid population from the cross IR64/Azucena (Courtois et al. 2000). The low correlation of RCGR with RWC_T and Score might be partly due to the complex genetic basic of RCGR, which is controlled by multiple genes and affected by other traits more than by leaf water status, and partly due to the low precision of the RCGR measurements requiring two destructive samplings, before and after stress, that involve different plants. In general, the phenotypic correlations between traits were quite similar in direction in the full panel, and in the indica and japonica subpanels. However, the japonica subpanel exhibited higher RWC and lower Score than indica subpanel in the first 3 weeks of drought stress, demonstrating significantly better drought tolerance. This may be due to the ecosystem origins of the japonica subpanel, in which approximately 50% of the accessions are upland varieties from the north mountainous regions, mainly belonging to population J1 and, to a lesser extent, to population J3 (Phung et al. 2014). By comparison, only 21% of the indica subpanel was composed of upland accessions (Phung et al. 2014). Upland rice varieties normally require less water compared to the other groups (Dingkuhn et al. 1991).

GWAS was performed for the two subpanels as well as for the full panel. Only one QTL (q3) was detected in the japonica subpanel, but this QTL was associated with RWC_T0, which was a trait nearly independent from the other traits and not affected by drought stress (Additional file 1: Table S7). The fact that no drought-tolerance associated QTL was detected in the japonica subpanel may be due to the small size of the japonica subpanel (64 accessions). A similar effect of the panel size was observed for root traits by Phung et al. (2016). Two traits, RCGR and RWC_S3, showed a very low heritability, and it is, therefore, not surprising that we could identify just one QTL for each of these traits and only in the full panel (i.e. q5 and q7, respectively). Of the 17 identified QTLs, 9 QTLs were detected for two or more traits (Table 4), which may suggest that these loci have pleiotropic effects on drought-related traits, while the 8 remaining loci were specific to different traits.

Most of the QTLs identified in this study co-localized with previously reported QTLs related to drought tolerance detected in bi-parental populations, and interestingly, some co-localizations were found for common drought-related traits (Additional file 1: Table S8). Among them, 8 QTLs shared similar traits (leaf rolling score, leaf drying score and leaf relative water content) with the QTLs discovered in our study (i.e. q1, q2, q4, q6, q7, q8, q9, and 13) (Hemamalini et al. 2000; Courtois et al. 2000; Price et al. 2002a; Gomez et al. 2006) (Additional file 1: Table S8). Interestingly, three QTLs (q6, q7, and q8) detected in this study and their co-localized QTL deriving from a double-haploid population (DH) (Hemamalini et al. 2000) were associated with leaf rolling score evaluated during the vegetative stage. Also at the vegetative stage, a co-location of q9 with the QTL identified by Price et al. (2002a) for leaf relative water content is identified. These co-localizations tend to confirm that our experimental conditions allowed the detection of genomic areas containing genetic determinants strongly involved in drought tolerance and suggest that the other QTLs identified in this study are potentially new QTLs determining drought-tolerance during the vegetative stage.

Overlaps of GWAS sites exhibited in our study and from previous GWAS studies were also observed, but at very low rate (3%) and without similar traits (Additional file 1: Table S9). This may be the consequence of differences in panels used, phenotyping conditions and traits monitored. The comparison between our GWAS for drought with the GWAS performed for root characters on the same Vietnamese rice landraces panel (Phung et al. 2016), shows no overlap between the associations identified for root and the drought tolerance characters (Additional file 5: Figure S4). This may be explained by differences in the two experimental systems: the root QTLs were detected in fully irrigated conditions in plants grown in long tubes allowing a full expression of root architecture parameters, while the QTLs in this study were identified in severe drought stress conditions applied to plants cultivated in pots, which did not fully allow expression of root adaptive characteristics. Nevertheless, scanning the qTARO database (http://qtaro.abr.affrc.go.jp/qtab/table) found that some of QTLs identified in this study were co-located with the previously reported QTLs associated with morpho-physiological traits and yield under drought stress in rice. For instance, the QTLs q1 and q2 in this study co-localized with QTLs associated with basal root thickness and root fresh weight in a DH population (Li et al. 2005). Another QTL from a DH population for total dry root weight (Nguyen et al. 2004) co-located with q11 detected in our study. q12 was found to be located within the mapped region of a QTL, qpl11.1, for yield and yield components detected in a BC2F2 population from an *Oryza sativa* x *Oryza rufipogon* cross (Moncada et al. 2001).

The identification below the most robust detected QTLs of 8 candidate genes whose reported function is related to drought and/or osmotic stress response and adaptation indicates that our study may be helpful for dissecting the genetic basis of drought tolerance in this panel. Some of these candidate genes are related to mechanisms that act at the cellular level to protect biochemical components against stress injury. In rice and other cereals, the increase in ferulic acid content was associated with the capacity of plants to tolerate water deficit. This compound was reported as one of the most effective photoprotectors that contribute to maintaining photosynthesis during drought stress (Hura et al. 2007; Ma et al. 2016). In this regard, *Os01g40870* that encodes an aldehyde dehydrogenase (*OsALDH2–2/ALDH1b*) and participates in the reaction of ferulic acid biosynthesis from coniferyl-aldehyde was proposed as a drought resistance candidate gene since it was differentially expressed during drought between two rice varieties contrasted for their water deficit tolerance (Ma et al. 2016). Aldehyde dehydrogenase gene overexpression can improve drought tolerance in *Arabidopsis thaliana* (Kotchoni et al. 2006; Chen et al. 2015). Similarly, *Os01g40860* encodes the aldehyde dehydrogenase *OsALDH2–1/ALDH1a*. Both genes are mainly expressed in roots and, to a lesser extent, in the leaf blade and sheath in normal conditions (RiceXpro, Li et al. 2000). Interestingly, *Os01g40870* (*OsALDH2–2/ALDH1b*) is located in a QTL for chlorophyll content under salt stress, *qChlo1*, and together with *Os01g4083*0, which is annotated as a transposon, constitute candidate genes for *qChlo1* based on the presence of non-synonymous SNP in their coding sequences (Panget al. 2017). In our study, these two genes are located within q1, which is highly associated with the drought sensitivity score at 3 weeks after drought treatment. It is likely that this gene plays a role in the adaptation of rice at the cellular level against osmotic stress. *Os05g25850* (*OsMSOD1/MnSOD*), located within q4, which is highly associated with the drought sensitivity score after 4 weeks of drought treatment, encodes a superoxide dismutase that is drought inducible, is more expressed in water deficit tolerant varieties and likely contributes to limit reactive oxygen species damage during drought stress (de Deus et al., 2015, Song et al. 2014). Increases in superoxide dismutase activity have been associated with drought tolerance capacity in several plant species (Turkan et al. 2005; Jagtap and Bhargava 1995; Wang et al. 2009). Located within q6 (associated with RWC_T4 and RWC_S4), *Os07g30590* encodes *OsLTPG20*, a non-specific lipid transfer protein with a GPI-anchor motif in the C-terminal region, which attaches the protein to the exterior side of the plasma membrane. Co-expression analysis suggested that these enzymes could contribute to the synthesis or deposition of cuticular waxes and suberin (Edstam et al. 2013). Such modifications of the external cell walls could contribute to a better adaptation to water deficit.

Other detected candidate genes concern signal transduction and transcription regulation. *Os11g12530*, a leucine-rich repeat receptor-like protein kinase (LRR-RLK), is located within q14, which is highly associated with the RWC as well as RWC_S at an early drought stage (T1). Some LRR-RLKs, such as FON1 (Feng et al. 2014), LP2 (Wu et al. 2015a, b), OsSIK1 (Ouyang et al. 2010), MsSIK1 (Guo et al. 2016), were reported to be involved in plant responses to drought stress. Analysis of the metadata derived from NCBI GEO expression datasets revealed that *Os11g12530* is predominantly expressed in roots and significantly induced by drought stress, but not up-regulated upon cold or submergence or in response to hormone treatments (ABA, IAA, JA) (Chandran et al. 2016).

*Os05g25770* encodes a WRKY45 transcription factor that is located within q4 (Score4). This gene is present in two allelic forms, specific to the japonica and indica subspecies, respectively. These alleles control differentially the response of the plant to abscisic acid and salt stress, but both alleles impact negatively on the capacity of the plant to tolerate water deficit (Tao et al. 2011).

q12 is not only highly associated with RWC_T3, as q1 is, but also strongly associated with RWC_T2. In plants, bZIP proteins play important roles in the ability to tolerate or adapt to abiotic stresses (Ji et al. 2009; E et al. 2014). *Os11g05480*, located within q12 and encoding *OsbZIP79*, which is a transcription factor, is down regulated by dehydration and salt stress but not by cold stress in 7-day-old rice seedlings (Nijhawan et al. 2008). This gene is also highly expressed in roots but less so in panicles and seedlings (Nijhawan et al. 2008). *OsbZIP79* can interact with *OsTGAP1*, another bZIP transcription factor that regulates the production of isoprenoid phytoalexins in roots, but unlike *OsTGAP1*, the overexpression of *OsbZIP79* causes the suppression of relevant gene expression for phytoalexin production in rice cells, which is a metabolite synthesized in response to pathogen infection (Miyamoto et al. 2015). As in the case of *Os11g05480*, *Os11g05640* encodes a transcription factor (*OsbZIP80*), which is also significantly up-regulated under dehydration stress, whereas it is down-regulated under cold and salt stress in 7-day-old seedlings (Nijhawan et al. 2008). Therefore, *OsbZIP80* was supposed to function as a dehydration stress-inducible gene in rice.

For the remaining QTLs, we found several genes which are probably also involved in abiotic response and drought signaling (Table 4).

To further validate the QTLs of interest identified in this study, it will be interesting to develop bi-parental segregating populations using contrasted varieties as parents. Moreover, identified candidate genes can be functionally tested by generating CRISPR-cas9 knock-out mutants. This material should allow for evaluating their contribution to drought tolerance-related traits. The favorable alleles of the validated genes will be used for marker-assisted introgression to improve rice tolerance to drought, particularly in Vietnamese rice varieties cultivated in regions affected by drought.

## Additional files

Additional file 1: **Table S1.** List of the 190 accessions used in the experiment. **Table S2.** Experiment map. **Table S3.** Standard evaluation score (SES) of drought sensitivity and recovery at vegetative stage (IRRI, 2002). **Table S4.** Abbreviations and full name of traits monitored in this study. **Table S5.** Descriptive statistics of the full panel for selected traits. RWC_T0, relative water content before drought treatment; RWC_T1 to RWC_T4, relative water content after 1 to 4 weeks of drought stress; RWC_S1 to RWC_S4, slope of relative water content after 1 to 4 weeks of drought stress; Score1 to Score4, drought sensitivity score after 1 to 4 weeks of drought stress; Recovery, recovery ability; RCGR, relative crop growth rate. **Table S6.** Correlation matrix between examined traits in the full panel computed by Pearson method (below the diagonal). Probabilities above the diagonal (in bold, significant at *P* < 0.05). F = full panel; I = indica subpanel; J = japonica subpanel). RWC_T0, relative water content before drought treatment; RWC_T1 to RWC_T4, relative water content after 1 to 4 weeks of drought stress; RWC_S1 to RWC_S4, slope of relative water content after 1 to 4 weeks of drought stress; Score1 to Score4, drought sensitivity score after 1 to 4 weeks of drought stress; Recovery, recovery ability; RCGR, relative crop growth rate. **Table S7.** Summary of genome-wide significant associations at *P* < 5e-04 for the full panel (F), the indica (I) and japonica (J) subpanels. RWC_T0, relative water content before drought treatment; RWC_T1 to RWC_T4, relative water content after 1 to 4 weeks of drought stress; RWC_S1 to RWC_S4, slope of relative water content after 1 to 4 weeks of drought stress; Score1 to Score4, drought sensitivity score after 1 to 4 weeks of drought stress; Recovery, recovery ability; RCGR, relative crop growth rate. **Table S8.** QTLs colocalized with the QTLs for drought-related traits detected in mapping populations from TropGeneDB. RWC_T0, relative water content before drought treatment; RWC_T1 to RWC_T4, relative water content after 1 to 4 weeks of drought stress; RWC_S1 to RWC_S4, slope of relative water content after 1 to 4 weeks of drought stress; Score1 to Score4, drought sensitivity score after 1 to 4 weeks of drought stress; Recovery, recovery ability; RCGR, relative crop growth rate (Lafitte and Courtois 1999; Price et al. 2002b). **Table S9.** Overlaps of detected associations with known GWAS-derived associations. (XLSX 196 kb)

Additional file 2: **Figure S1.** Effect of drought on vegetative growth of a panel of rice Vietnamese landraces. A, before stress treatment; B, two weeks after drought treatment; C, four weeks after drought treatment; D, two weeks after rewatering. (PDF 482 kb)

Additional file 3: **Figure S2.** Correlations between traits in the indica and japonica subpanels. RWC_T0, relative water content before drought treatment; RWC_T1 to RWC_T4, relative water content after 1 to 4 weeks of drought stress; RWC_S1 to RWC_S4, slope of relative water content after 1 to 4 weeks of drought stress; Score1 to Score4, drought sensitivity score after 1 to 4 weeks of drought stress; Recovery, recovery ability; RCGR, relative crop growth rate. (PDF 213 kb)

Additional file 4: **Figure S3.** Manhattan plots for genome-wide association of drought-related traits with SNP markers for the indica and japonica subpanels. A (RWC_T3), relative water content after 3 weeks of drought stress; B (RWC_T2), relative water content after 2 weeks of drought stress; C (RWC_S2), slope of relative water content after 2 weeks of drought stress; D (Score3), drought sensitivity score after 3 weeks of drought stress; E (Recovery), recovery ability; F (Score1), drought sensitivity score after one week of drought stress; G (Score2), drought sensitivity score after 2 weeks of drought stress; H (RWC_S4), slope of relative water content after 4 weeks of drought stress; I (RWC_T4), relative water content after 4 weeks of drought stress; J (RWC_S1), slope of relative water content after one week of drought stress; L (RWC_T1), relative water content after one week of drought stress; M (Score4), drought sensitivity score after 4 weeks of drought stress; N (RCGR), Relative crop growth rate; O (RWC_S3), slope of relative water content after 3 weeks of drought stress. (PDF 899 kb)

Additional file 5: **Figure S4.** Map position of detected QTLs (in green) with QTLs for root traits (Phung et al. 2016) (in purple) using the same panel of Vietnamese rice landraces and genotyping data. RWC_T0, relative water content before drought treatment; RWC_T1 to RWC_T4, relative water content after 1 to 4 weeks of drought stress; RWC_S1 to RWC_S4, slope of relative water content after 1 to 4 weeks of drought stress; Score1 to Score4, drought sensitivity score after 1 to 4 weeks of drought stress; Recovery, recovery ability; RCGR, relative crop growth rate. LLGTH, longest leaf length; TIL, number of tillers; SDW, shoot dry weight; DEPTH, deepest point reached by roots; MRL, maximum root length; NCR, number of crown roots; NR_T, number of crown root per tiller; THK, root thickness; DW0020, root mass in the 00–20 cm segment; DW2040, root mass in the 20–40 cm segment; DW4060, root mass in the 40–60 cm segment; DWB60, root mass below 60 cm; DRW, deep root mass (< 40 cm) weight; RDW, root dry weight; PDW, plant dry weight; SRP, shallow root proportion (0–20 cm); DRP, deep root proportion (< 40 cm); R_S, root to shoot ratio. (PDF 243 kb)
