# Supplementary figures and images for: Genome-wide Association Study of a Panel of Vietnamese Rice Landraces Reveals New QTLs for Tolerance to Water Deficit During the Vegetative Phase
Source: Rice (N Y). 2019 Jan 28;12:4. doi: 10.1186/s12284-018-0258-6 (PMC6357217; doi:10.1186/s12284-018-0258-6)

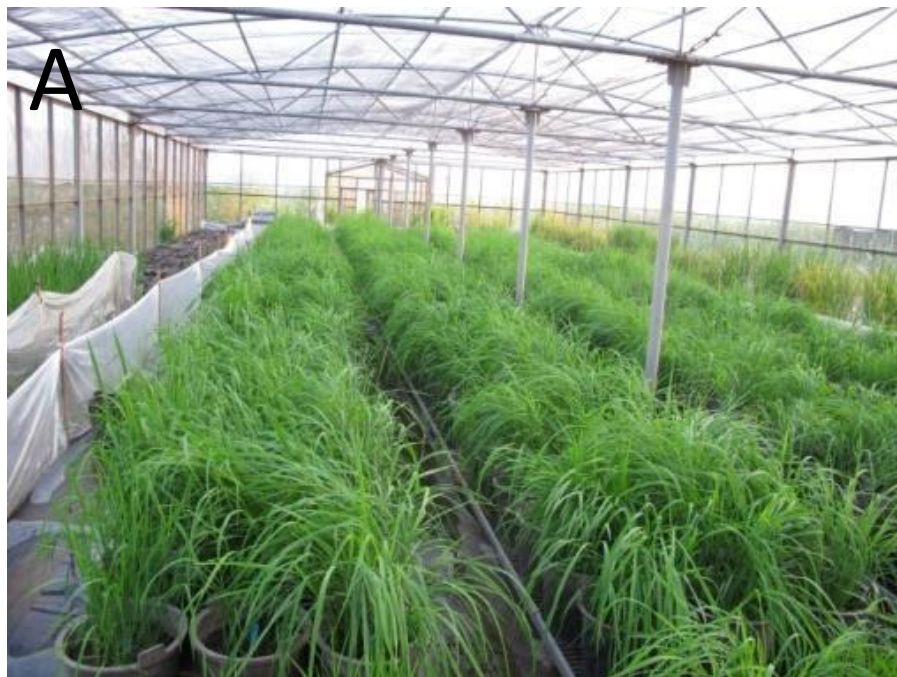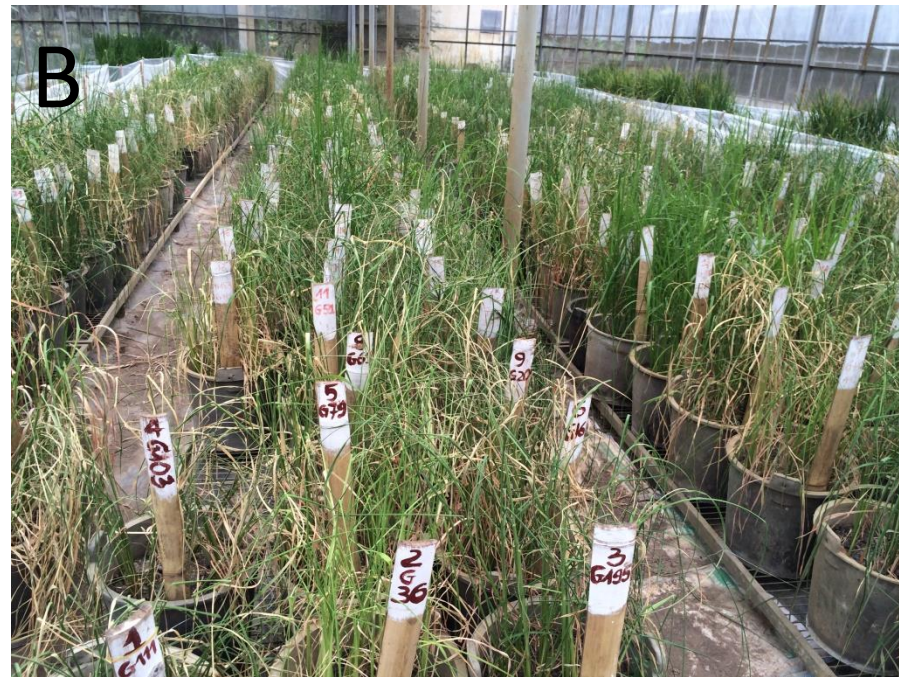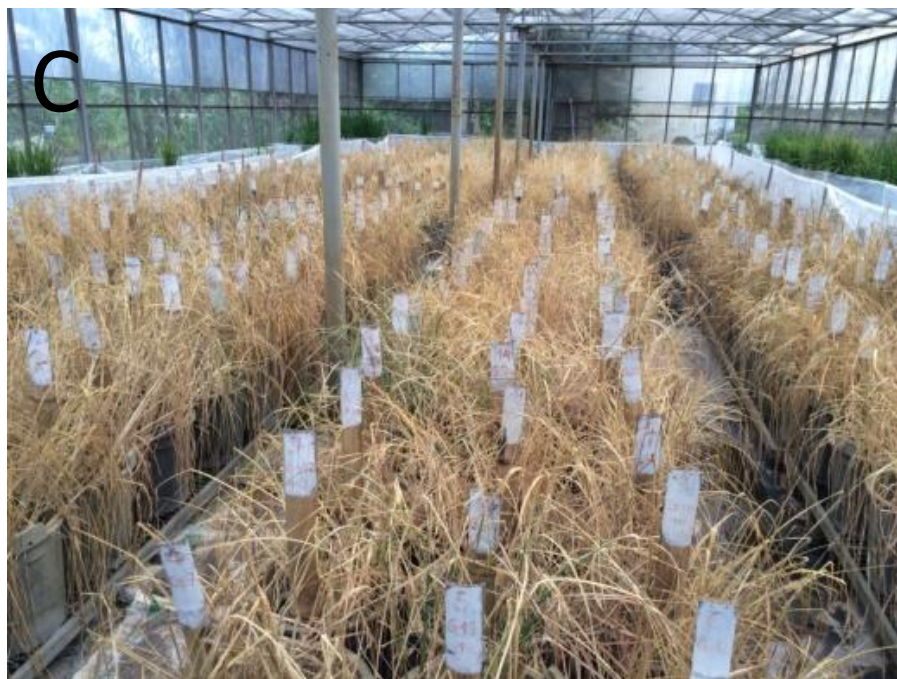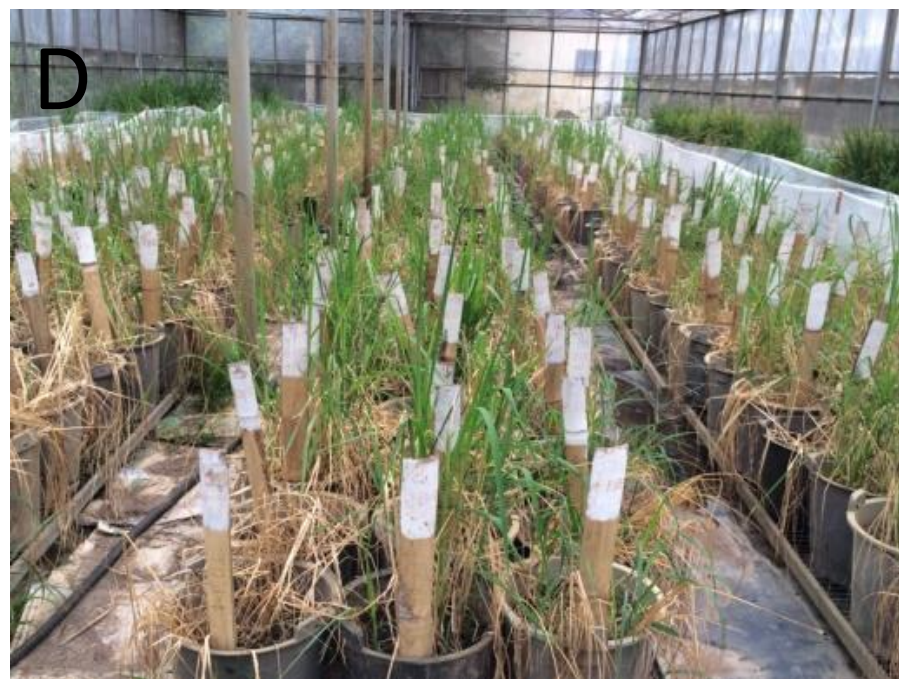

Supplement: Supplementary file 2 — Figure S1. Effect of drought on vegetative growth of a panel of rice Vietnamese landraces. A, before stress treatment; B, two weeks after drought treatment; C, four weeks after drought treatment; D, two weeks after rewatering. (PDF 482 kb) [file 12284_2018_258_MOESM2_ESM.pdf]

## *indica*

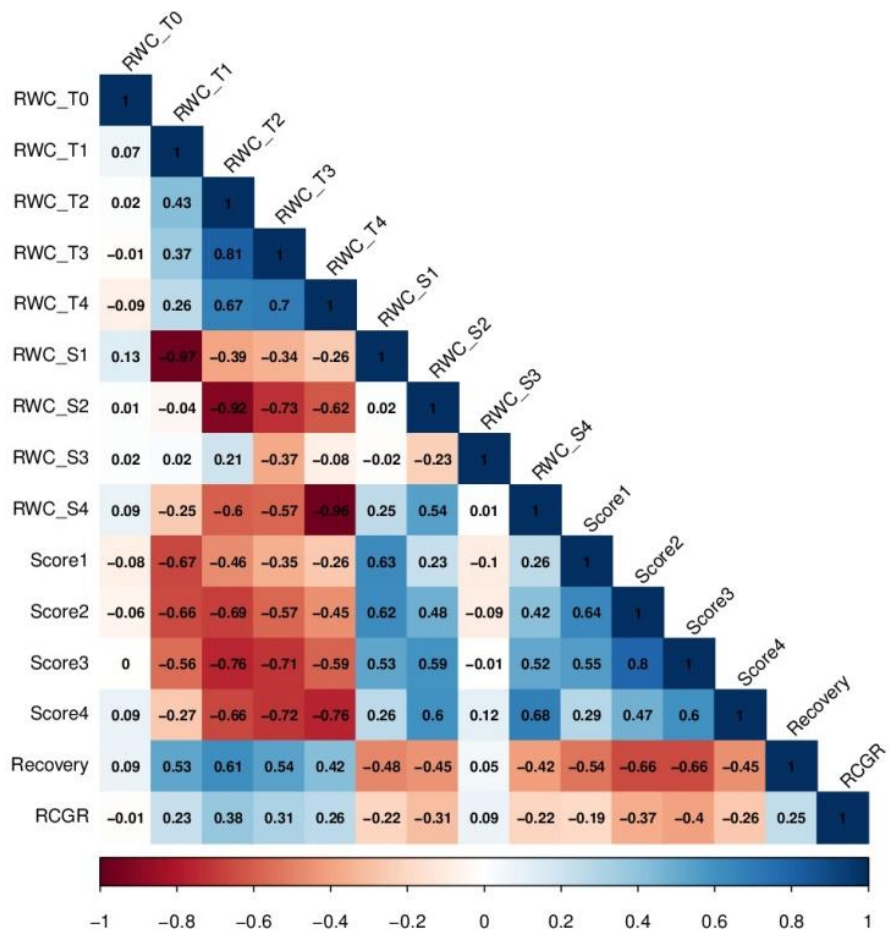

## *japonica*

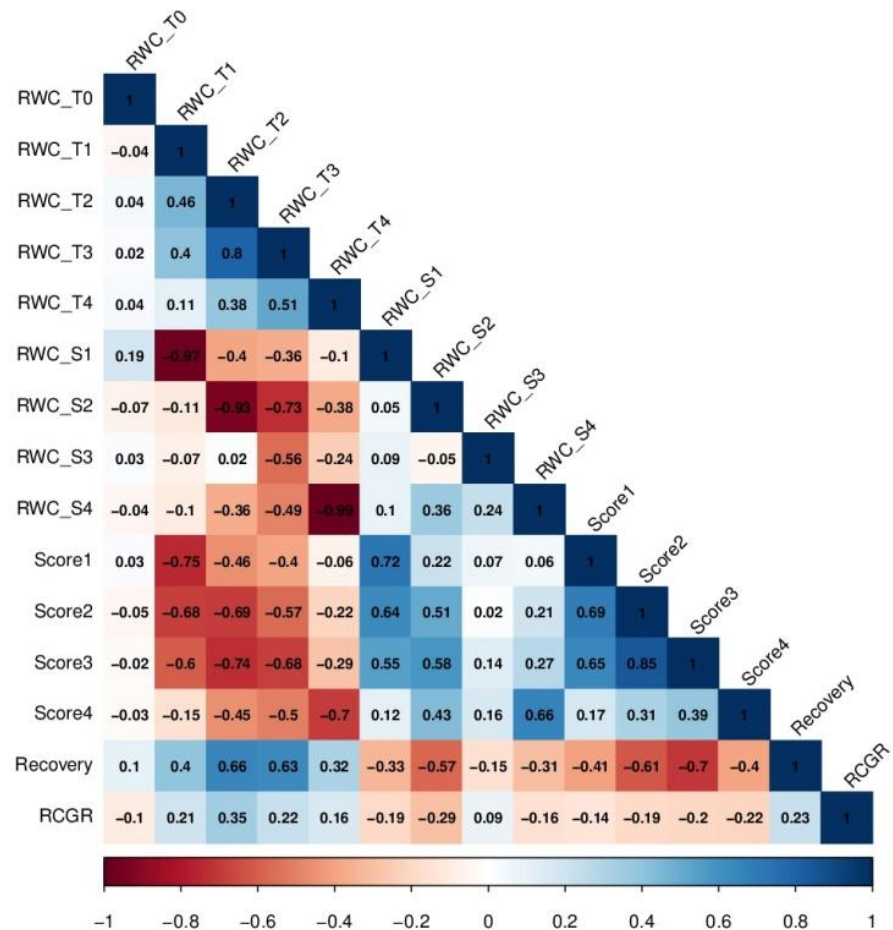

Supplement: Supplementary file 3 — Figure S2. Correlations between traits in the indica and japonica subpanels. RWC_T0, relative water content before drought treatment; RWC_T1 to RWC_T4, relative water content after 1 to 4 weeks of drought stress; RWC_S1 to RWC_S4, slope of relative water content after 1 to 4 weeks of drought stress; Score1 to Score4, drought sensitivity score after 1 to 4 weeks of drought stress; Recovery, recovery ability; RCGR, relative crop growth rate. (PDF 213 kb) [file 12284_2018_258_MOESM3_ESM.pdf]

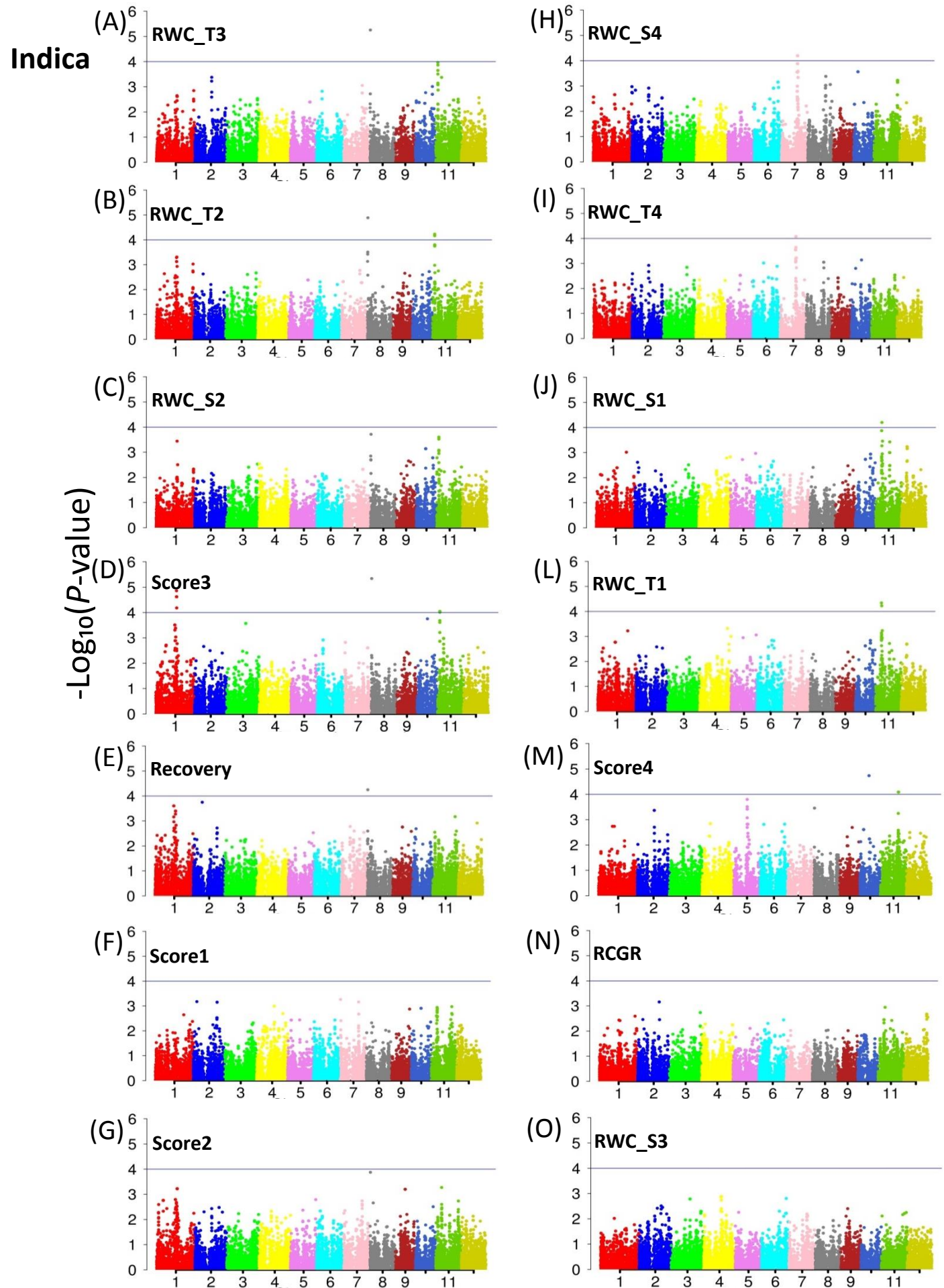

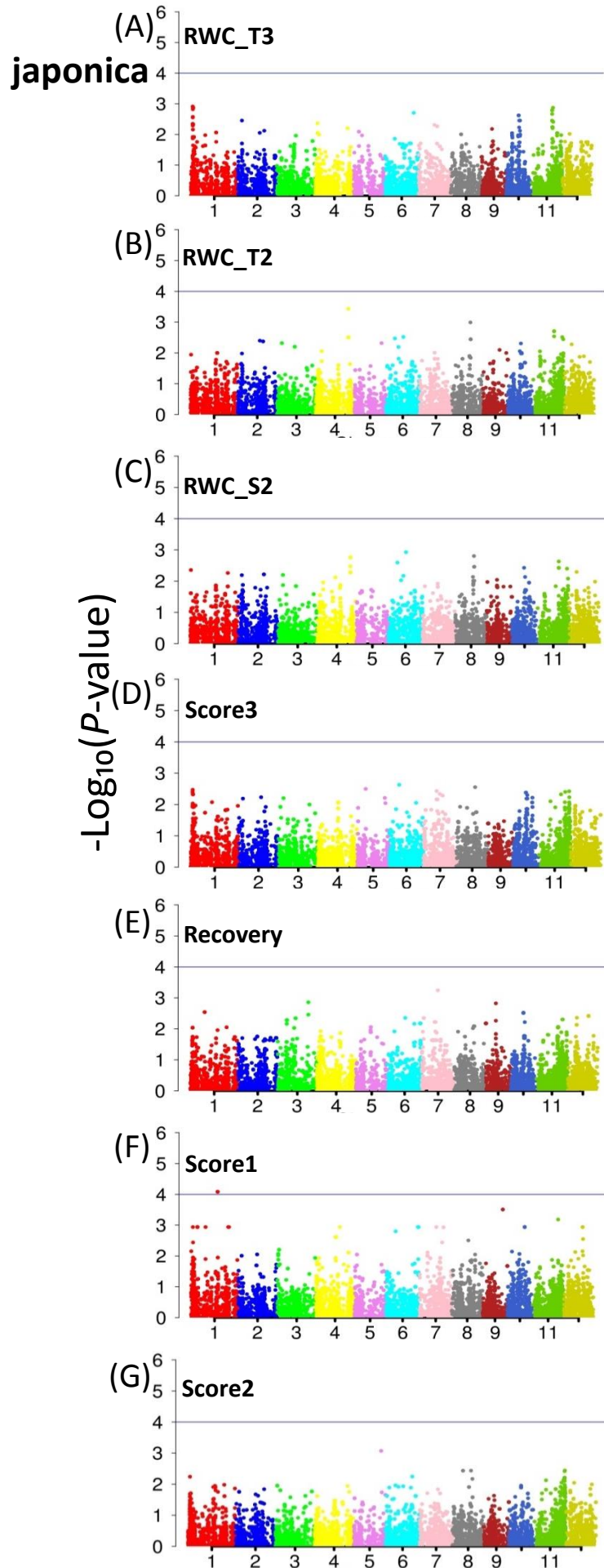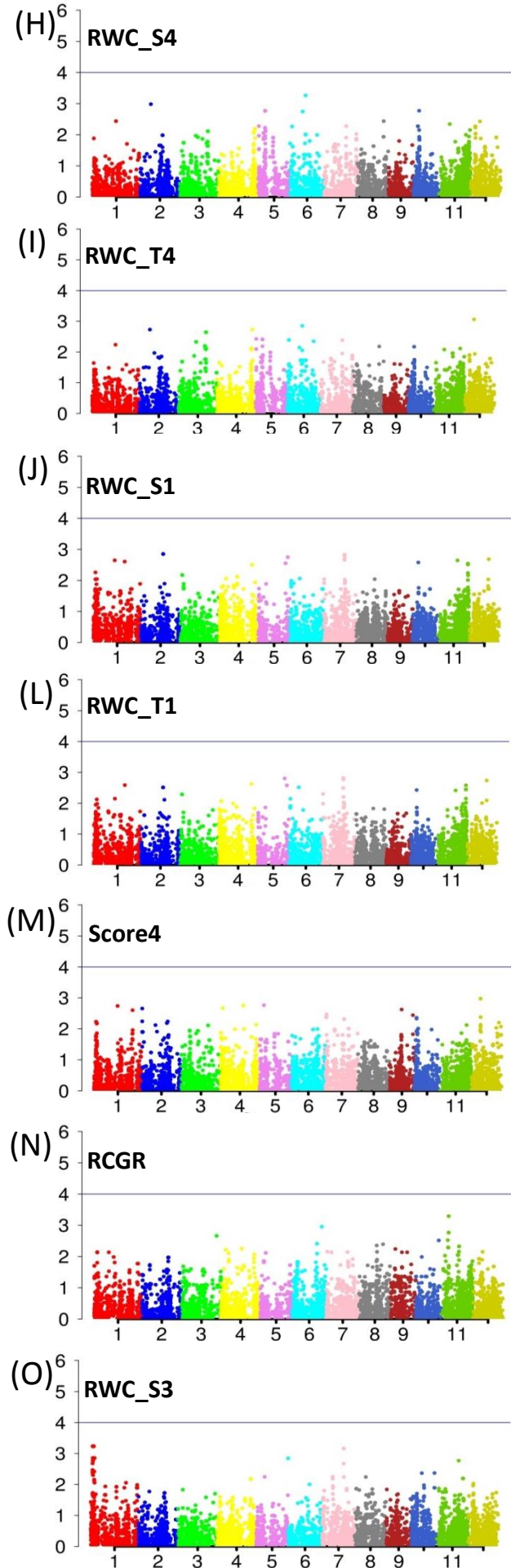

Supplement: Supplementary file 4 — Figure S3. Manhattan plots for genome-wide association of drought-related traits with SNP markers for the indica and japonica subpanels. A (RWC_T3), relative water content after 3 weeks of drought stress; B (RWC_T2), relative water content after 2 weeks of drought stress; C (RWC_S2), slope of relative water content after 2 weeks of drought stress; D (Score3), drought sensitivity score after 3 weeks of drought stress; E (Recovery), recovery ability; F (Score1), drought sensitivity score after one week of drought stress; G (Score2), drought sensitivity score after 2 weeks of drought stress; H (RWC_S4), slope of relative water content after 4 weeks of drought stress; I (RWC_T4), relative water content after 4 weeks of drought stress; J (RWC_S1), slope of relative water content after one week of drought stress; L (RWC_T1), relative water content after one week of drought stress; M (Score4), drought sensitivity score after 4 weeks of drought stress; N (RCGR), Relative crop growth rate; O (RWC_S3), slope of relative water content after 3 weeks of drought stress. (PDF 899 kb) [file 12284_2018_258_MOESM4_ESM.pdf]

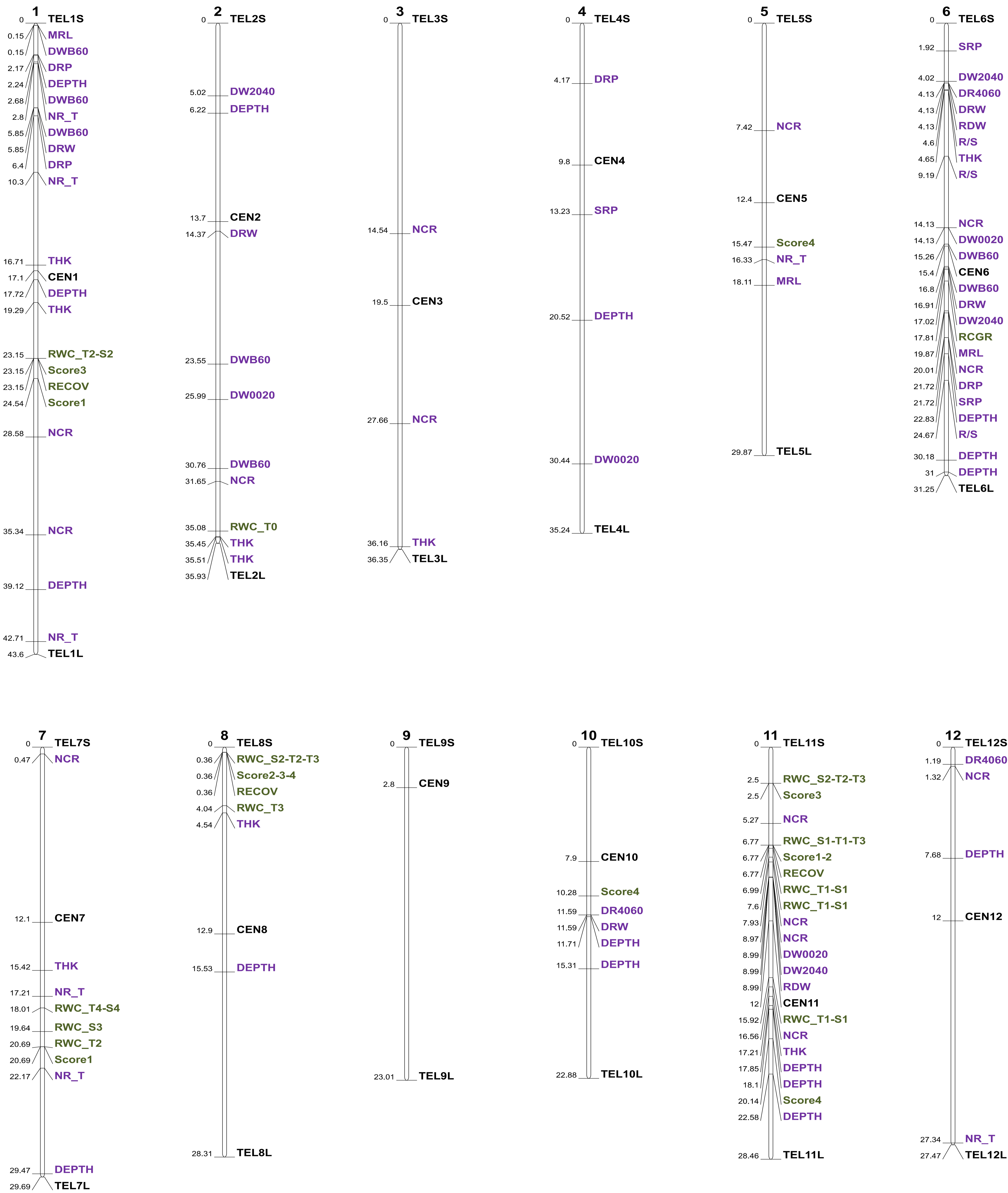

Supplement: Supplementary file 5 — Figure S4. Map position of detected QTLs (in green) with QTLs for root traits (Phung et al. 2016) (in purple) using the same panel of Vietnamese rice landraces and genotyping data. RWC_T0, relative water content before drought treatment; RWC_T1 to RWC_T4, relative water content after 1 to 4 weeks of drought stress; RWC_S1 to RWC_S4, slope of relative water content after 1 to 4 weeks of drought stress; Score1 to Score4, drought sensitivity score after 1 to 4 weeks of drought stress; Recovery, recovery ability; RCGR, relative crop growth rate. LLGTH, longest leaf length; TIL, number of tillers; SDW, shoot dry weight; DEPTH, deepest point reached by roots; MRL, maximum root length; NCR, number of crown roots; NR_T, number of crown root per tiller; THK, root thickness; DW0020, root mass in the 00–20 cm segment; DW2040, root mass in the 20–40 cm segment; DW4060, root mass in the 40–60 cm segment; DWB60, root mass below 60 cm; DRW, deep root mass (< 40 cm) weight; RDW, root dry weight; PDW, plant dry weight; SRP, shallow root proportion (0–20 cm); DRP, deep root proportion (< 40 cm); R_S, root to shoot ratio. (PDF 243 kb) [file 12284_2018_258_MOESM5_ESM.pdf]
